# Matrine combined with Osthole inhibited the PERK apoptosis of splenic lymphocytes in PCV2-infected mice model

**DOI:** 10.1186/s12917-023-03581-9

**Published:** 2023-01-30

**Authors:** Yinlan Xu, Shuangxiu Wan, Panpan Sun, Ajab Khan, Jianhua Guo, Xiaozhong Zheng, Yaogui Sun, Kuohai Fan, Wei Yin, Hongquan Li, Na Sun

**Affiliations:** 1 Shanxi Key Lab. for Modernization of TCVM, College of Veterinary Medicine, Shanxi Agricultural University, Taigu, 030801 Shanxi China; 2 School of Public Health, Xinxiang Medical University, Xinxiang, 453003 Henan China; 3 School of Pharmacy, Heze University, Heze, 274000 Shandong China; 4 Department of Veterinary Pathology, Faculty of Veterinary and Animal Sciences, the University of Agriculture, Dera Ismail Khan 29050, Khyber Pakhtunkhwa, Pakistan; 5 Department of Veterinary Pathobiology, Schubot Exotic Bird Health Center, Texas A&M University, College Station, Texas, TX 77843 USA; 6 Medical Research Council (MRC) Centre for Inflammation Research, Queen’s Medical Research Institute, The University of Edinburgh, Edinburgh, EH16 4TJ UK; 7 Laboratory Animal Center, Shanxi Agricultural University, Taigu, 030801 Shanxi China

**Keywords:** Matrine, Osthole, PCV2, GRP78, Apoptosis, PERK

## Abstract

**Background:**

Porcine circovirus type 2 (PCV2) is one of the major pathogens commonly found in pigs, which causes immunosuppression and apoptosis. Vaccination and a single drug cannot totally prevent and treat PCV2 infection. Our previous in vitro study reported that the synergistic anti-PCV2 effect of Matrine and Osthole was better than that of Matrine or Osthole alone, This study was aimed to evaluate the synergistic anti-PCV2 effect as well as the underline molecular mechanism of Matrine and Osthole in Kunming (KM) mice model infected with PCV2. KM mice were randomly divided into 8 groups namely control group, PCV2 infected, Matrine combined with Osthole high dose treatment (40 mg/kg + 12 mg/kg), medium dose treatment (20 mg/kg + 6 mg/kg), low dose treatment (10 mg/kg + 3 mg/kg), Matrine treatment (40 mg/kg), Osthole treatment (12 mg/kg) and Ribavirin positive control (40 mg/kg) groups. PCV2 was intraperitoneally (i.p.) injected in all mice except the control group. 5 days of post-infection (dpi), mice in different treatment groups were injected i.p. with various doses of Matrine, Osthole and Ribavirin once daily for the next 5 consecutive days.

**Results:**

The synergistic inhibitory effect of Matrine and Osthole on PCV2 replication in mouse liver was significantly heigher than that of Matrine and Osthole alone. The expression of GRP78, p-PERK, p-eIF2α, ATF4, CHOP, cleaved caspase-3 and Bax proteins were significantly reduced, while that of Bcl-2 was significantly increased in Matrine combined with Osthole groups, which alleviated the pathological changes caused by PCV2, such as interstitial pneumonia, loss of spleen lymphocytes, infiltration of macrophages and eosinophils.

**Conclusions:**

The synergistic anti-apoptotic effect of Matrine and Osthole was better than their alone effect, Both Matrine and Osthole had directly inhibited the expression of PCV2 Cap and the apoptosis of spleen cells induced by PCV2 Cap through the PERK pathway activated by endoplasmic reticulum (ER) GRP78. These results provided a new insight to control PCV2 infection and provide good component prescription candidate for the development of novel anti-PCV2 drugs.

**Supplementary Information:**

The online version contains supplementary material available at 10.1186/s12917-023-03581-9.

## Background

PCV2 is an important pathogen of Post-weaning Multisystemic Wasting Syndrome (PMWS), inducing immunosuppression and apoptosis in the infetced piglet [1], and has become one of the most economically important diseases to the global pig industry [2]. The PCV2 vaccines effectively reduced the virus infection in pigs since it was widely used in 2007. However, the PCV2 strain is prone to mutation and cannot be best matched with existing vaccines, resulting in the inability of vaccine immunity to eradicate PCV2 infection [3, 4]. Sometimes this mutation is accelerated [5], and therefore, there is an urgent need to develop anti-PCV2 drugs to prevent and mitigate the injury. Due to the high efficacy and safety level, low toxicity, low residual level, as well as unique clinical significance of natural compounds, researches are focused to investigate the anti-viral activities of these compounds. PCV2 is a DNA virus causing immunosuppression. Many viruses have developed complex mechanisms to interact and evade the host immune system providing cellular targets for antiviral intervention [6]. Endoplasmic reticulum (ER) is an important site for viral replication and maturation. The up-regulation of molecular chaperone GRP78 is an ER stress marker [7]. According to Zhou et al. [8] PK-15 cells infected with PCV2 causes endoplasmic reticulum stress (ERS), induce the expression of stress marker protein GRP78, initiate the unfolded protein response to selectively activate the PERK/eIF2a/ATF4/CHOP apoptotic pathway to induce apoptosis in PK-15 cells.

Matrine is an alkaloid and the main bioactive component of *sophora*. It has anti-inflammatory [9], anti-cancer [10], anti-virus [11, 12], anti-apoptosis [13, 14], and other biological activities. Sun et al. [15] proved that Matrine possessed antiviral activities via inhibiting virus replication and regulating immune functions in mice co-infected with PRRSV/PCV2. Liu et al. [16] confirmed that Matrine can significantly reduce the expression of inflammatory genes in mice with liver or lung injuries in the treatment of novel Coronavirus through real-time RT-PCR. The results suggest that Matrine can simultaneously intervene the combination of COVID-19 and liver injury through multiple pharmacological mechanisms.

Osthole is the main bioactive ingredient of *Fructus Cnidii* and has anti-inflammatory [17], anti-cancer [18], anti-virus [19], anti-immunosuppression [20] and anti-apoptosis effects [21]. Huang et al. [19] proved that Osthole increases glycosylation of hepatitis B surface antigen and suppresses the secretion of hepatitis B virus in vitro. Studies have shown that osthole has anti-TMV (Tobacco Mosaic Virus) activity and may be used as a biological reagent to control the plant virus in the half-leaf method [22]. However, no literature has been found on the antiviral effect of osthole in animal models. Our previous studies have shown that Matrine combined with Osthole has a synergistic anti-PCV2 and anti-apoptosis effects induced by PCV2 Cap in vitro [13]. However, due to complex clinical treatment mechanisms and animal body functions, the in vivo anti-PCV2 effect and the underline mechanism of Matrine combined with Osthole are still unclear.

**Fig. 1:**
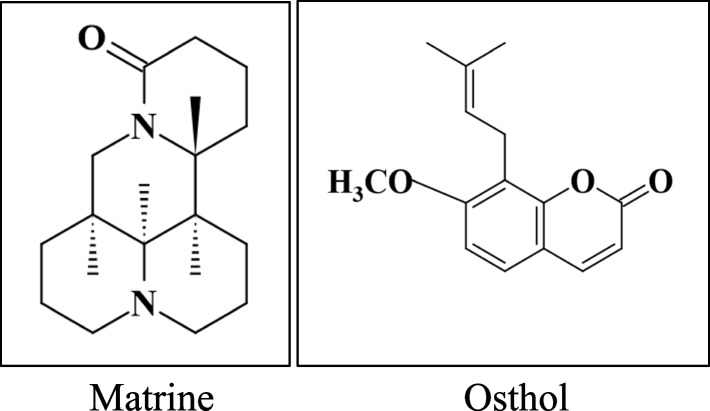
Chemical structure of matrine and Osthole

Therefore, as the active ingredients of Traditional Chinese medicine, Matrine and Osthole were selected according to the guidance of traditional Chinese veterinary theories to formulate a component prescription with clear chemical structure, purity, specific biological and various pharmacological activities (The chemical structure of Matrine and Osthole was shown in Fig. 1). Moreover, to explore the molecular synergistic anti-PCV2 effect of Matrine combined with Osthole in a PCV2 infected mouse model.

## Results

### Establishment of KM mice model infected with PCV2

**Fig. 2:**
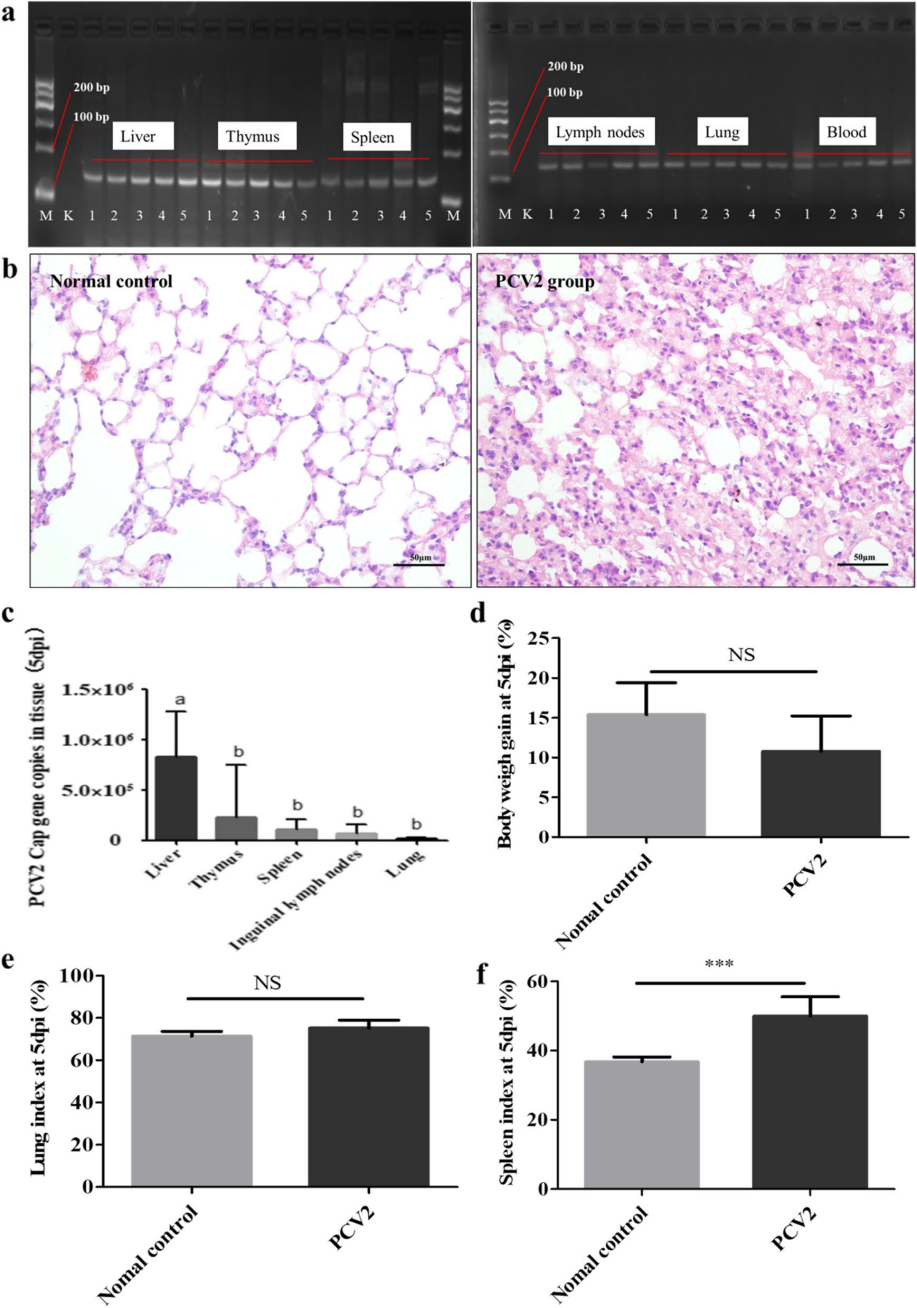
Establishment of the model of KM mice infected with PCV2. **a** The *Cap* gene in tissues and blood of mice was determined after 5dpi. with PCV2 by PCR. M indicated the Marker, K indicated the normal mice and mumber 1–5 indicated individual PCV2 infected mouse. **b** HE staining results of the mice lungs showed that, compared with the normal group, the pulmonary interstitium of the PCV2 group was significantly widened and the alveolar cavity was significantly reduced. **c** The qPCR results showed that the highest expression of *Cap* gene in the liver was obsevered. **d-f** body weight gain, lung index and spleen index. Compared with the normal control group, there was no significant difference in the weight gain rate and lung index in the PCV2 infection group, while the spleen index increased significantly. ***means *p* < 0.001. Different letters indicated significant differences between the groups, *p* < 0.05

PCR results showed that compared with the control mice group, PCV2 *Cap* gene were detected in the liver, thymus, spleen, inguinal lymph nodes, lung and blood of mice at 5 dpi of PCV2 (Fig. 2a, the original images see Additional file 1). The results indicated that the KM mouse model of PCV2 infection was successfully established and the whole body of the mice was in the state of viral infection.

Compared with the control group, H&E staining elucidated that the lungs of mice infected with PCV2 at 5^th^ dpi showed typical features of interstitial pneumonia including significant widened interstitium and reduced alveolar space (Fig. 2b, the original images see Additional file 1). Therefore, the lungs were used to evaluate the effect of Matrine combined with Osthole on the pathological injury of mice infected with PCV2 in subsequent experiments. The qPCR results showed the expression of *Cap* gene in the liver, thymus, lungs, spleen and lymph nodes of mice infected with PCV2 with highest expression of *Cap* gene in the liver (*P* < 0.05) as shown in Fig. 2c. Therefore, liver was selected as a target organ and the expression levels of PCV2 *Cap* gene and protein were analysed for the assessment of synergistic anti-PCV2 effect of Matrine and Osthole.

It was revealed that there was no significant difference in body weight gain and lung index between control and PCV2 challenged mice (*P* > 0.05). However, the spleen index in PCV2 challenged mice was significantly increased, compared with the control group (*P* < 0.05) (Figs. 2d-f). Therefore, mice spleen was used as a target organ to investigate the synergistic effect of Matrine and Osthole on the pathological changes caused by PCV2 infection and the mechanism of anti-PCV2.

### PCV2 replication in mouse liver

**Fig. 3:**
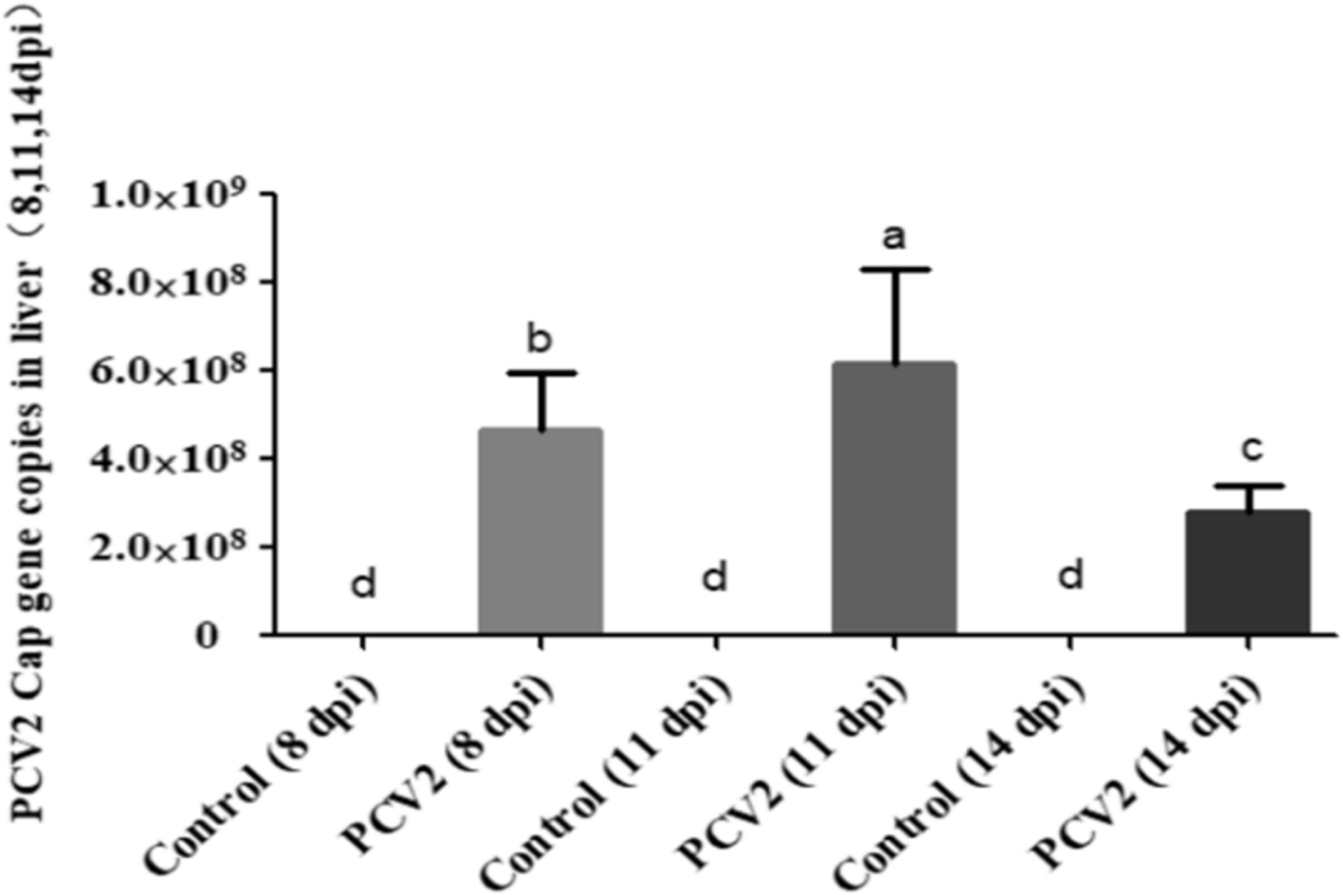
The replication of PCV2 *Cap* gene in mouse liver. The expression of PCV2 *Cap* gene in the liver of mice infected by PCV2 at 8 d, 11 d and 14 d was detected by qPCR. Different letters indicate significant differences between groups, *p* < 0.05

In order to explore the PCV2 replication in mouse, qPCR was used to detect the expression of *Cap* genes in mice liver infected with PCV2 at 8, 11 and 14 days were detetmined by qPCR. The results showed that the viral load in liver of the PCV2 infected group was the highest at 11 d (6 d after compounds adminstration) of infection with PCV2 (Fig. 3). It was therefore concluded these results provided a base for selecting the time points to study the synergistic anti-PCV2 effects of Matrine combined with Osthole.

### Inhibition of the PCV2 replication in mouse liver

The expression of *Cap* gene in the liver of PCV2 infected mice at 8, 11 and 14 days (3, 6 and 9 after compounds adminstration) was detected by qPCR.

**Fig. 4:**
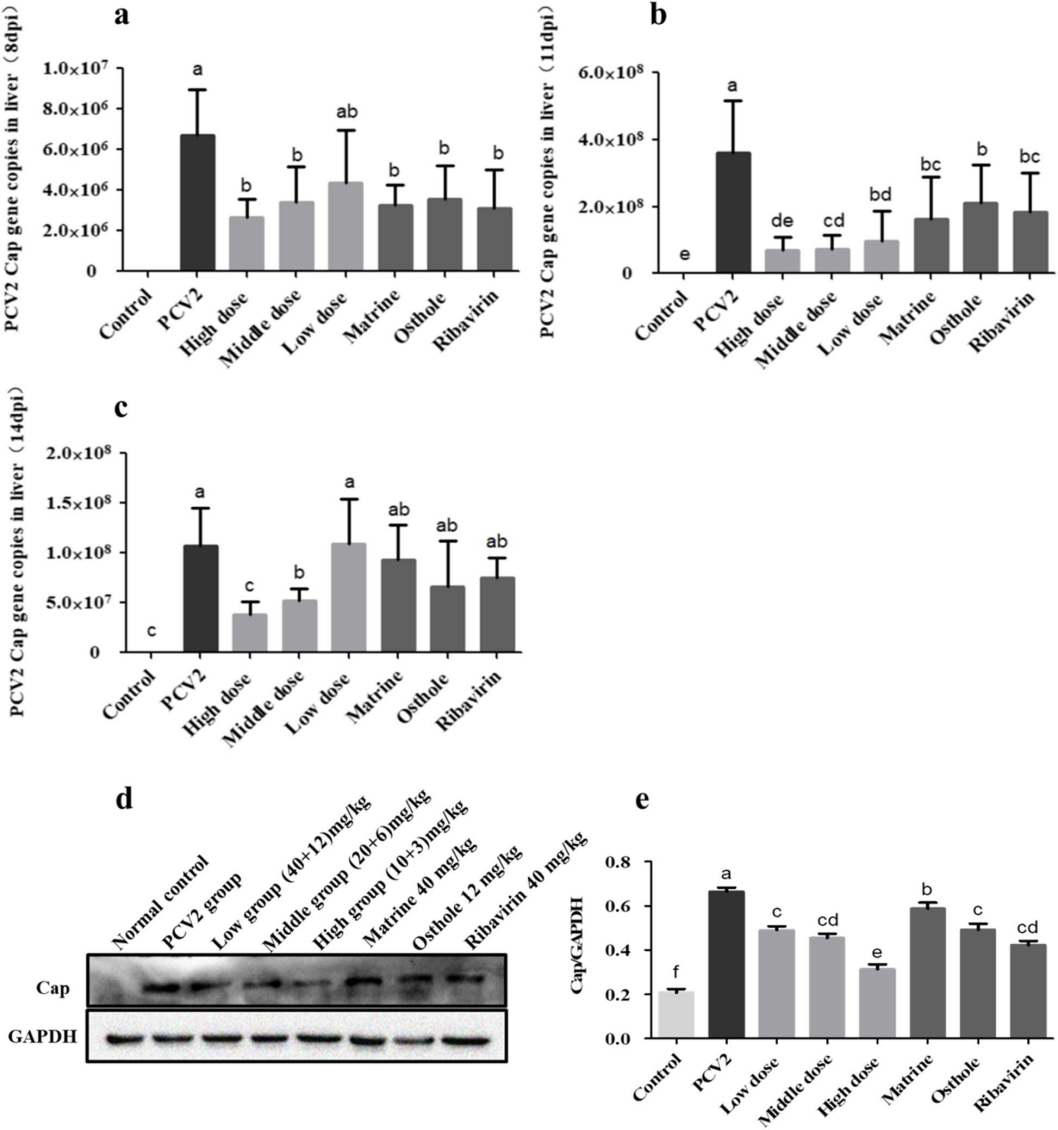
Inhibited the expression of PCV2 *Cap* gene and protein in mouse liver by Matrine combined with Osthole. **a-c** Expression of PCV2 *Cap* gene in mouse liver at d 8, 11 and 14 (3 d, 6 d and 9 after compounds adminstration) of Matrine combined with Osthole was detected by qPCR, respectively. Data were showed as copy number. **d** and **e** Expression of PCV2 Cap protein in mice liver treated for 11 d with the combined of Matrine and Osthole. Representative western blot image and the data of calculated Cap/GAPDH ratio were showed. Cropped blots are displayed. Different letters indicated significant differences between the groups (*p* < 0.05)

Compared with the control group, the results showed that the expression of *Cap* genes was significantly increased in the PCV2 infected group(*p* < 0.05) as shown in Figs. 4a-c. Compared with the PCV2 untreated group, the expression of *Cap* gene was significantly reduced in the high dose (40 mg/kg + 12 mg/kg) and medium dose (20 mg/kg + 6 mg/kg) of combined groups (Fis. 4a-c). Similarly, compared with the Matrine, Osthole and Ribavirin alone groups, the results of PCV2 infected mice at 8 d showed no significant difference in the expression of *Cap* genes in the combined high, medium and low dose groups (Fig. 4a, *p* < 0.05). But at 11 d the expression of *Cap* gene was significantly reduced in the high and medium dose combined groups compared with the Matrine and Osthole alone groups (*p* < 0.05), and no significant difference in the expression of *Cap* genes in the low dose combined group (Fig. 4b, *P* > 0.05). At 14 d, compared with the Matrine, Osthole and Ribavirin alone groups, the expression of *Cap* gene was significantly reduced in all combined groups except for the low dose one (Fig. 4c, *p* < 0.05).

In conclusion, the replication of PCV2 Cap gene in the mice liver were inhibited by the combined treatment of Matrine and Osthole. In addition, the results in Fig. 3 showed that “the viral load of PCV2 on the infected mouse liver was highest at 11 d post infection”. Therefore, mice infected with PCV2 for 11 days (6 d after compounds adminstration) were selected for subsequent studies.

Compared with the PCV2 infection untreated group, Figs. 4d and 4e (the original blot images involved see Additional file 2.) showed that, the expression of Cap proteins in all treatment groups were significantly reduced (*P* < 0.05). The expression of Cap proteins was significantly lowered in the high dose combined group than Matrine, Osthole and Ribavirin alone groups (*p* < 0.05). Compared with the Matrine group of 40 mg/kg, the expression of Cap protein was significantly reduced in the medium and low dose combined groups (*P* < 0.05), but no significant change was found when compared with the Osthole and Ribavirin alone groups (*P* > 0.05).

In short, the synergistic anti-PCV2 effect of Matrine combined with Osthole at 40 mg/kg + 12 mg/kg was demonstrated.

### The effect of Matrine combined with Osthole on PCV2 infected lungs and spleen

**Fig. 5:**
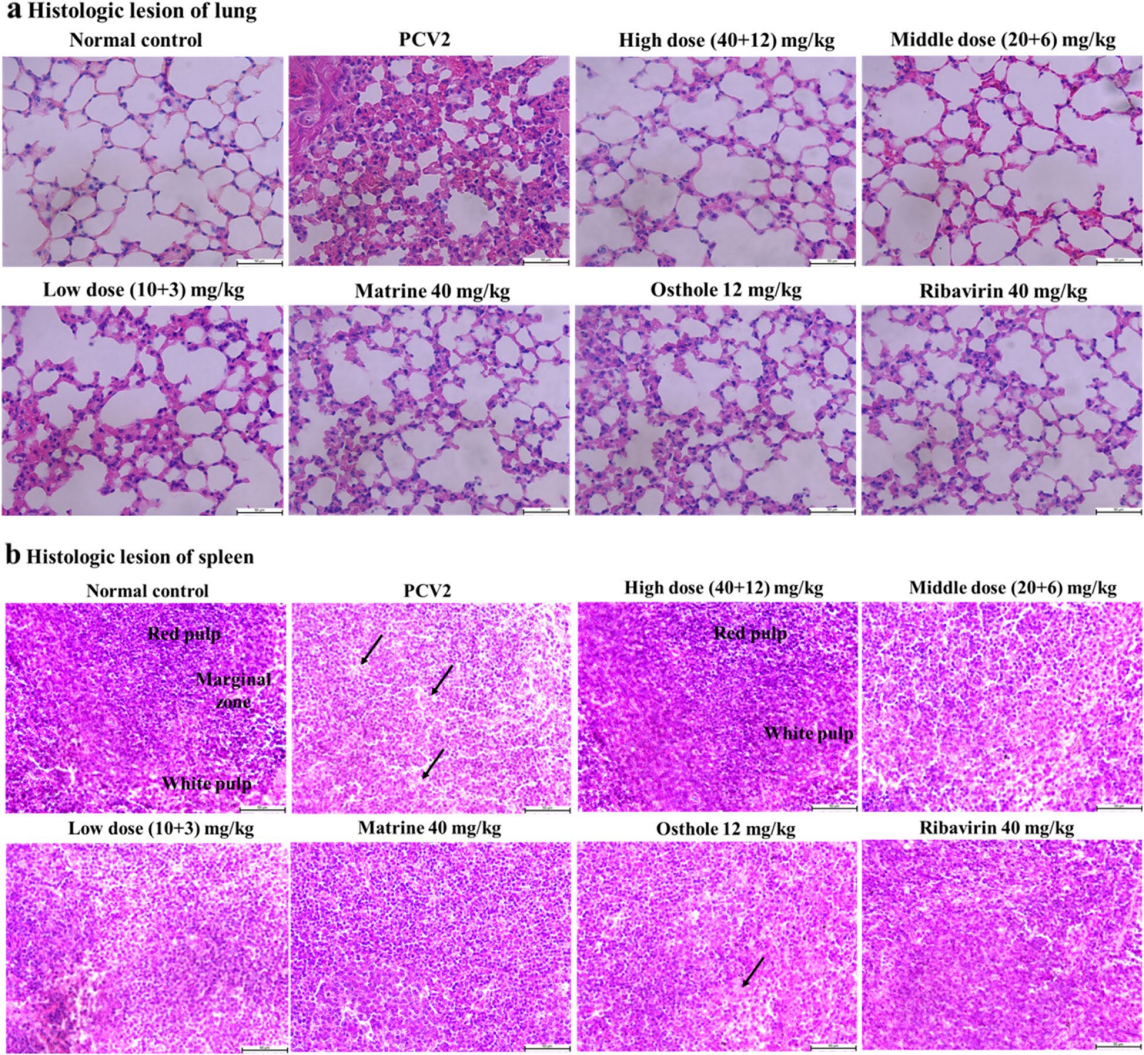
Effect of Matrine combined with Osthole on pathological change of lung and spleen induced by PCV2 infection. Representative tissue histology images of mice at 11 d (6 d after compounds adminstration) after PCV2 infection were showed. Sale bar = 50 μm. **a** Results of lungs HE staining demonstrated that compared with normal control, PCV2 group displayed severe thickened alveoli septum and alveolar space decreased. **b** The rusults of spleen HE staining showed that loss of lymphocytes and infiltration of macrophages and eosinophils in the PCV2 group While the pathological changes of each treatment group were significantly alleviated, especially in the group with Matrine combined with Osthole was the best

Representative images of H&E stained lungs and spleen tissues of mice infected with PCV2 at 11 d after PCV2 infection were shown in Fig. 5. Compared with the lungs of mice in the control group, the alveolar septum in the PCV2 group was widened and the alveolar cavity was significantly narrowed indicating the typical symptoms of interstitial pneumonia. The pulmonary pathological changes induced by PCV2 was alleviated in all treated groups, among which Matrine and Osthole combined treatment showed better effect (Fig. 5a, the original HE staining images see Additional file 3). The deletion of lymphocytes and infiltration of macrophages and eosinophil was observed in the spleen of PCV2 infected mice as compared with the control mice. The pathological changes induced by PCV2 in spleen was improved significantly in all treatments groups, especially in the Matrine and Osthole combined treatment group (Fig. 5b, the original HE staining images see Additional file 4).

### Matrine combined with Osthole inhibited the apoptosis of spleen lymphocytes in PCV2 infected mice

**Fig. 6:**
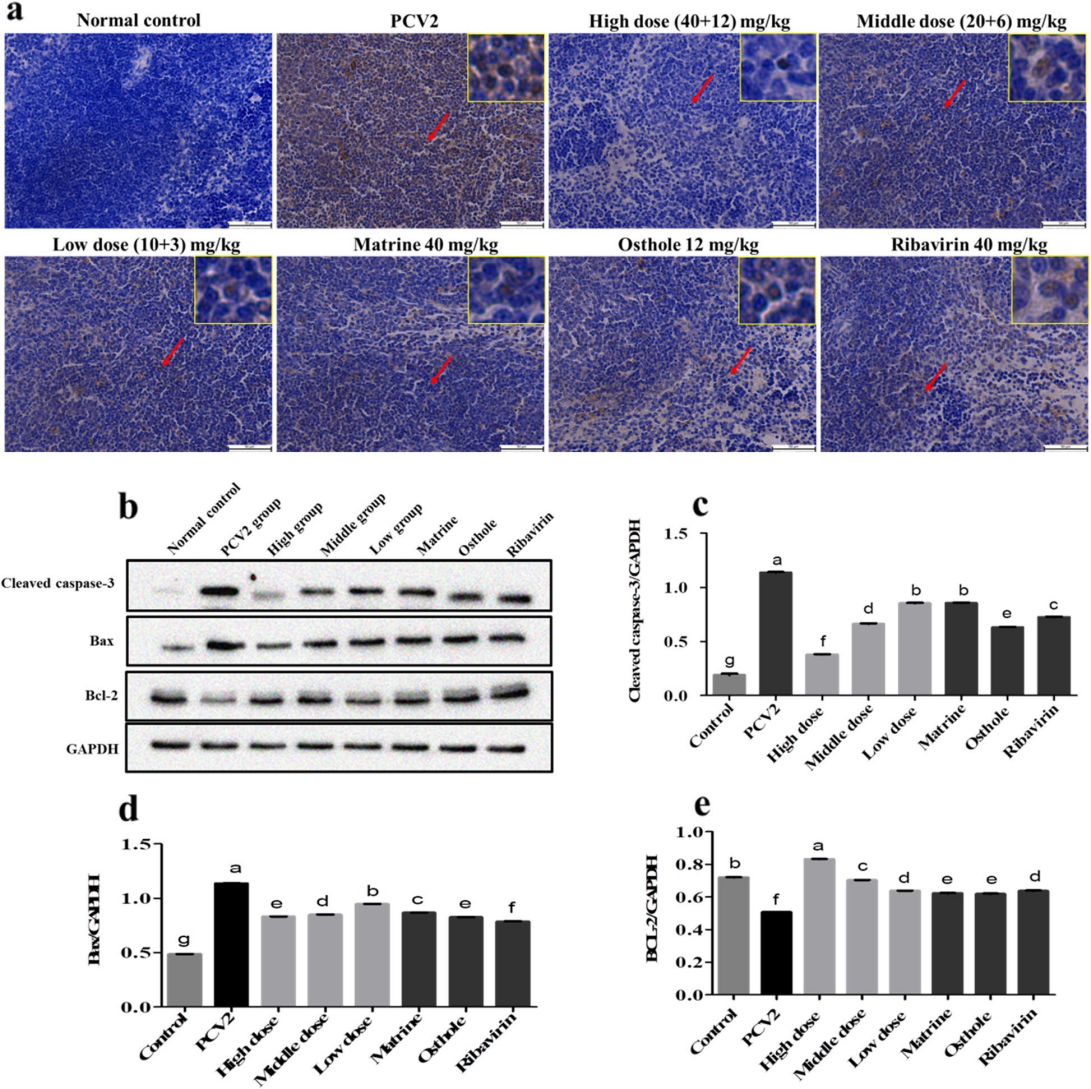
Inhibited the apoptosis of spleen lymphocytes in KM mice infected with PCV2 at 11 d by Matrine combined with Osthole. **a** The IHC results of spleen showed that, compared with the normal group, the apoptosis of lymphocytes in the spleen tissue was more significant in the group of PCV2 infection group. The cell apoptosis was inhibited in each treatment group, and the group of Matrine combined with Osthole had the strongest effect (Bar = 50 μm). **b-e** Western blot results showed that the expression of cleaved caspase-3 and Bax was significantly increased (*p* < 0.05) and the expression of Bcl-2 was significantly decreased (*p* < 0.05). Cropped blots are displayed, the samples derive from the same experiment and that gels/blots were processed in parallel. Different letters indicated significant differences between the groups, *p* < 0.05

The results of IHC showed that the expression of cleaved caspase-3 protein in the spleen was increased in the PCV2 group at 11 d (6 d after compounds adminstration) of PCV2 infection as compared with that in the control group. Compared with the PCV2 untreated group, the expression of cleaved caspase-3 apoptin in all treatment groups was decreased (Fig. 6a, the original IHC images involved see Additional file 5).

The results of Western blot showed that, compared with the control, the expressions of cleaved caspase-3 and Bax were significantly increased (*p* < 0.05) while the expression of Bcl-2 was significantly decreased in PCV2 infected mice at 11 d (*p* < 0.05). Compared with the PCV2 group, the expression of cleaved caspase-3 and Bax in all treated groups were significantly reduced (*p* < 0.05), but the expression of Bcl-2 was significantly increased (*p* < 0.05). The expression of cleaved caspase-3 was significantly reduced and that of Bcl-2 was significantly increased in the high dose combined group as compared with the Matrine, Osthole and Ribavirin alone groups (*p* < 0.05). The expression of Bax in the high dose combined group was significantly reduced as compared with the Matrine and Ribavirin alone groups (*p* < 0.05), but no significant difference was found with the Osthole alone group (*p* > 0.05) (Figs. 6b-e, the original blot images involved see Additional file 6).

### Inhibition of spleenic lymphocytes apoptosis through the PERK pathway mediated by GRP78

**Fig. 7:**
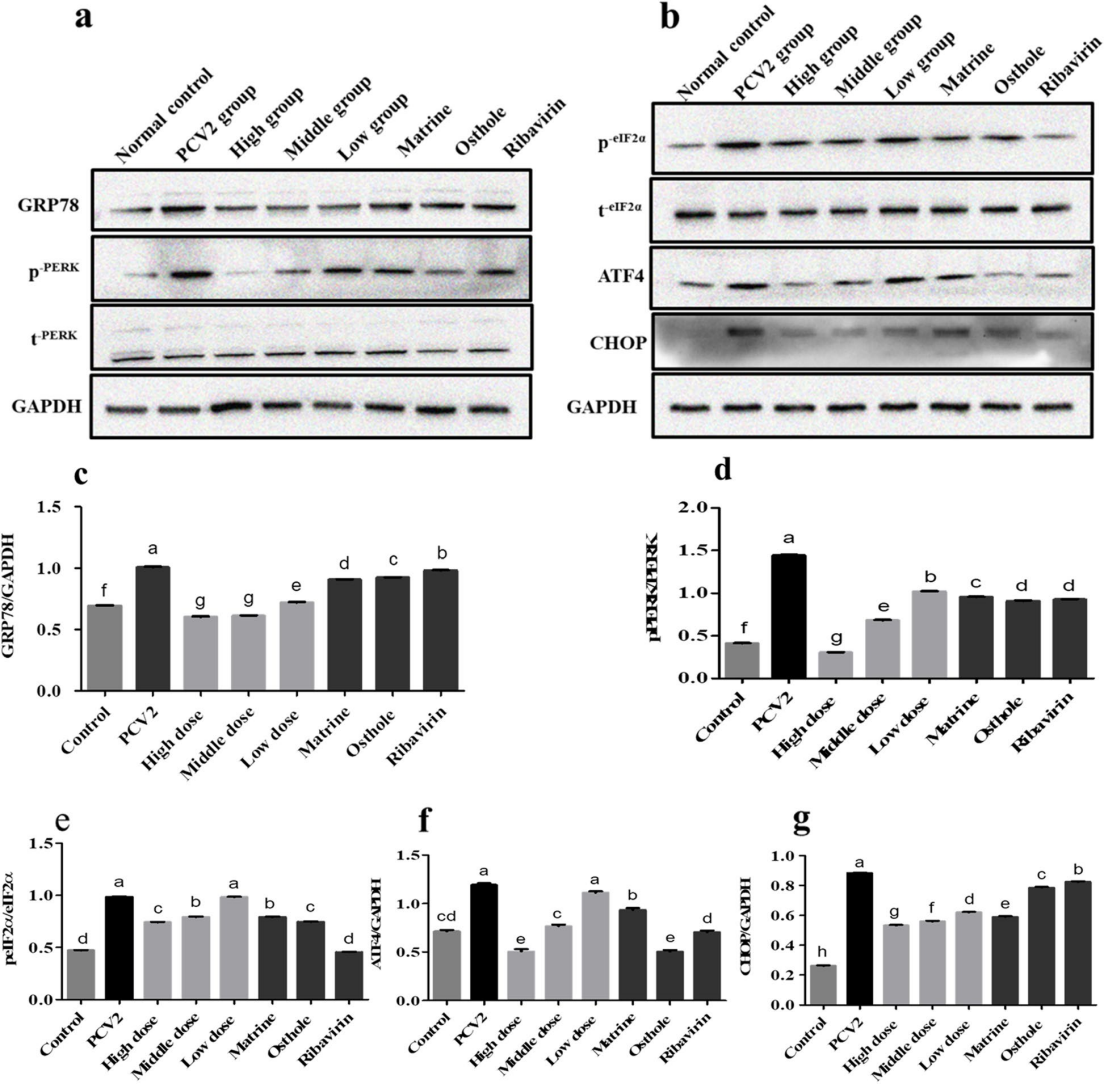
The apoptosis of spleen lymphocytes in PCV2 infected mice was inhibited by Matrine combined with Osthole through the PERK pathway. **a-g** The expression levels of GRP78 and each apoptin in the PERK pathway were analyzed by Western blot and grayscale analysis. After being treated with Matrine combined with Osthole, the levels of GRP78, p-PERK, p-eIF2α, ATF4 and CHOP were down-regulated. Cropped blots are displayed, the samples derive from the same experiment and that gels/blots were processed in parallel. Data are expressed in the form of Mean ± SEM, Different letters indicated significant differences among groups, *p* < 0.05

Compared with the control group, the results of Western blot (Fig. 7, for the original blot images see Additional file 6) showed that the expressions of pro-apoptins GPR78, p-PERK, p-eIF2α, ATF4 and CHOP were significantly increased in the PCV2 infected mice at 11 d (*p* < 0.05). Compared with the PCV2 untreated group, the expression of GPR78, p-PERK, p-eIF2α, ATF4 and CHOP was significantly reduced in the high and medium dose combined groups (*p* < 0.05). Compared with the groups of Matrine, Osthole and Ribavirin alone groups, the expression of GRP78 was significantly reduced in the high, medium and low dose combined groups (*p* < 0.05), while that of p-PERK and CHOP were significantly reduced in the high and medium dose combined groups. The expression of p-eIF2α was significantly reduced in the high dose combination group as compared with the Matrine treated group but no significant difference was found compared with the Osthole group (*p* < 0.05).

In summary, the apoptosis of mice spleen lymphocytes induced by PCV2 were significantly inhibited by the high dose and medium dose of Matrine and Osthole combined treatment through the PERK/eIF2α/ATF4/CHOP pathway activated by GRP78 (*p* < 0.05), although Matrine, Osthole and Ribavirin alone also have the inhibitory effect.

## Discussion

PCV2 is an important pathogen that causes Post-weaning Multisystemic Wasting Syndrome (PMWS) in weaned piglets, which is also known as PCV2-associated diseases (PCVAD) [2]. The virus replicates in multiple tissues of piglets such as liver, kidney, lung, spleen, thymus and lymph nodes, causing interstitial pneumonia and immunosuppression [23]. PCV2 preferentially targets the lymphoid tissues which leads to immunosuppression of pigs. Post infection of lymphoid tissue, a large number of histiocytes and multinucleated giant cell infiltration are observed with decrease and disappearance of lymphocytes [24]. PCV2 can also replicate in the lungs, liver, spleen and other tissues of KM mice after infection with PCV2, but it mainly causes interstitial pneumonia and immunosuppression [25]. Although mice are not the natural hosts of PCV2, apoptosis of macrophages was reported to be detected in the spleen of mice infected with PCV2 [26]. Experiments in laboratory are the gold standard for evaluating the pharmacology of drugs [6]. Therefore, this study evaluated the synergistic anti-PCV2 effects of Matrine combined with Osthole on the established PCV2 infected KM mouse model to replace the pig model.

In this study, KM mice infected with PCV2 showed signs of lung interstitial pneumonia, partial lymphocyte loss and macrophage infiltration in the spleen, and higest expression of *Cap* gene in the liver. This conclusion is consistent with the previous reports [27] in which piglets infected with PCV2 causes lymphocyte loss and monocytes infiltration. The Pathological effects of spleen shown in H&E staining are also consistent with the PCV2 infected laiwu pigs (LW) and Yorkshire pigs (YL) [28]. Some literature demonstarated that the typical symptoms of interstitial pneumonia was observed in piglets [29] and KM mice [25] infected with PCV2. The findings of this stusy is consistent with the above mentioned reports. Yan et al. [30] reported that Matrine and astragalus polysaccharides had a synergisticly protective effect on ulcerative colitis and associated lung injury in rats. Matrine combined with APS exerted a better protective effect against UC and lung injury compared with the treatments of APS and matrine alone groups. It was reported that Matrine combined with Docetaxel had a synergistic effect on prostate cancer than the other lone treatment groups [31]. Jarząb et al. [32] reported that Osthole and Cisplatin in combination augment their anti-cancer activities and yielded an additive type of pharmacologic interaction by means of isobolographic analysis. This study revealed that the replication of PCV2 in mouse liver was synergisticly inhibited by the combined use of Matrine and Osthole, which was consistent with the our previous in vitro results that Matrine combined with Osthole had synergistic anti-PCV2 effects [13]. In addition, the results of our study showed that the antiviral effect of matrine and osthole in combination was better than that of the Matrine and Osthole alone treatment with a dose-dependent nature, which was consistent with the results reported in the literature [30–32].

ER is an important site for virus replication and maturation. Upregulation of molecular chaperone GRP78 is a sign of ER stress. GRP78 can activate the pathways of IRE1, ATF6 and PERK [7], and PCV2 could selectively activate the PERK pathway induced by GRP78, while cannot activate the IRE1a/XBP1 and ATF6 pathways [8]. ER stress leads PERK to become an active kinase with the ability to phosphorylate α subunits of the eIF2, which induces the preferential translation of ATF4. The ATF4 then activates CHOP to promote apoptosis [33]. The results of IHC and Western blot of mice spleen infected with PCV2 at 11 d post infection showed apoptosis of spleen lymphocytes, which was consistent with the previous reaserch finding [26].

In this study, the expression of apoptin was detected in the spleen of mice by Western blotting. PCV2 Cap target GPR78, activate PERK apoptosis pathway and induce apoptosis of spleen lymphocytes in PCV2 infected mice. Moreover, the PERK pathway activated by GRP78 was inhibited by the combined use of Matrine and Osthole. The combination treatment inhibited the apoptosis of mouse spleen lymphocytes induced by PCV2, indicating that GRP78 may be a potential target for compound treatment of PCV2 infection in vivo. Zhou et al. [8] reported that in PK-15 cells infected with PCV2, PCV2 Cap caused ERS, promoted the apoptosis of PK-15 cells by GRP78 induced PERK/eIF2a/ATF4/CHOP apoptosis pathway. The results of our study are consistent with the results of Zhou's study, because both suggest that Grp78 is a potential therapeutic target.

Gao et al. [34] demonstrated that the expression of GRP78, p-PERK, p-IRE1α and ATF6α in ER pathway was regulated by Matrine and improved ER stress and mitochondrial dysfunction caused by non-alcoholic fatty liver disease. Therefore, GRP78 is an important target for the development of antiapoptotic drugs.

## Conclusions

**Fig. 8:**
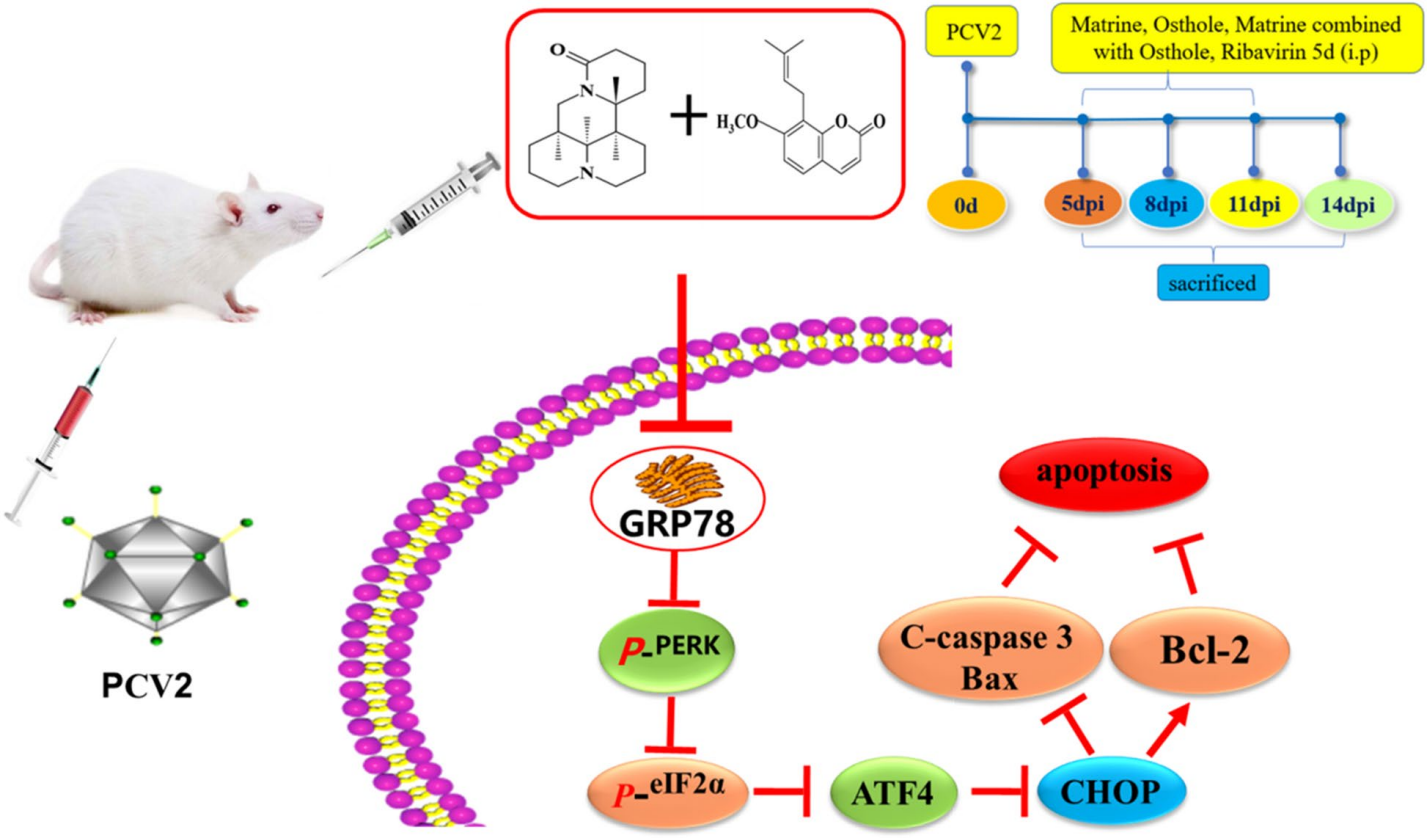
Mechanism of anti-apoptosis of spleen lymphocytes induced by PCV2 using Matrine combined with Osthole via GRP78-mediated PERK/eIF2α/ATF4/CHOP/ Bcl-2 pathway. The “T” and “arrow” indicate inhibition and promotion, respectively

Both Matrine and Osthole have anti-PCV2 effect, and inhibit PCV2-induced apoptosis by suppressing PERK/eIF2α/ATF4/CHOP/ Bcl-2 pathway mediated by GRP78 (Fig. 8). The synergistic inhibitory effect of Matrine combined with Osthole was much stronger than that of the two alone. The results provided a new prospect for further investigation of anti-PCV2 infection, and are expected to provide good prescription candidate for the development of novel anti-PCV2 used GRP78 as the therapeutic target.

## Methods

### PCV2, compounds and antibody

PCV2-SH strain was provided by professor Jiang Ping of Nanjing Aricultural University. PK-15 cells were used for virus propagation. The virus titer of PCV2 was 10^6.4^ TCID_50_ /mL, which was determined by indirect immunofluorescence assay (IFA).

The chemical structure of Matrine and Osthole was shown in Fig. 1. Matrine with 98.7% purity was purchased from National Institutes for Food and Drug Control (lot number:110805–201,709). The maximum safe concentration (MSC) used in this experiment was 40 mg/kg [15]. Osthole with 98.0% purity was purchased from Nanjing Zelang Biotechnology Co., Ltd, China (Lot number:ZL20171212SCZS). Osthole was first dissolved in dimethyl sulfoxide (DMSO) and diluted to 12 mg/kg in normal saline, 12 mg/kg of Osthole did not shownhemolysis in hemolysis assay. In this experiment, three dose of Matrine combined with Osthole was used at 40 mg/kg + 12 mg/kg (combined high dose), 20 mg/kg + 6 mg/kg (combined medium dose) and 10 mg/kg + 3 mg/kg (combined low dose). KM mice were monitored for 14 d after intraperitoneal injection. The mice administrated Matrine combined with Osthole were healthy. Therefore, the combined dose was considered to be safe for KM mice. Ribavirin with 99.0% purity, the positive control drug, was purchased from Beijing Solarbio Technology Co., Ltd, China (CAS: 36791–04-5), the MSC used in this experiment was 40 mg/kg [15].

Antibodies against Cap and p-PERK were purchased from Biorbyt LLC. (1:1000; California, Britain) and immunology Biology Technology Co., Ltd. (1:1000; Beijing, China), respectively. GRP78 (1:1000), PERK (1:800), eIF2α (1:800), p-eIF2α (1:500), ATF4 (1:800), CHOP (1:1000), Bcl-2 (1:1000), Bax (1:800) and cleaved caspase-3 (1:1000) were purchased from abcam (Cambridge, USA). GAPDH and horseradish peroxidase (HRP)-conjugated secondary antibodies were purchased from Wuhan Sanying Biology Technology Co., Ltd.(1:5000; Wu Han, China)and ComWin Biotech Co., Ltd. (1:15,000; Beijing, China), respectively.

### KM mice and administration

All KM mice used in this study was humanely managed according to the animal use protocol approved by the Animal Ethical Committee of the Shanxi Agricultural University. SPF female mice (18–20 g) were purchased from Beijing Vital River Laboratory Animal Technology Co., Ltd. Animal license number is SCXK (Beijing) 2016–0011. Mice were maintained under a 12:12 light/dark cycle with temperature (22 ± 1℃), humidity (60 ± 10%) and receving food and water ad libitum.

One hundred and fifty-four (154) KM mice were randomly divided into 8 groups after acclimation (Table 1): control, PCV2 infected control, combined high dose treatment groups (40 mg/kg + 12 mg/kg), combined medium dose treatment groups (20 mg/kg + 6 mg/kg), combined low dose treatment groups (10 mg/kg + 3 mg/kg), Matrine treatment groups (40 mg/kg), Osthole treatment groups (12 mg/kg) and Ribavirin treatment positive control groups (40 mg/kg). 0.5 mL PCV2 with 10^5.4^ TCID_50_/mL was injected intra-peritonially in to mice and an equivalent amount of 0.9% saline was injected to the control group. The compounds was injected i.p. once daily for 5 consecutive days after the mice were infected with PCV2 for 5 days, at a dose of 0.2 mL/10 g, and the control group was given an equal amount of saline with 10% DMSO at the same frequency and by the same route. Ribavirin was used as the positive drug control group. All mice were weighed daily. The mice were euthanized at 5 d (0 d after compounds adminstration), 8 d (3 d after compounds adminstration), 11 d (5 d after compounds adminstration and without compounds adminstration for 1 d, designated 6 d after compounds adminstration) and 14 d after PCV2 infection (5 d after compounds adminstration and without compounds adminstration for 4 d, designated as 9 d after compounds adminstration). The blood was collected in the anticoagulant tube and stored temporarily in a refrigerator at 4℃. The liver and spleen were collected and quickly put into liquid nitrogen for cryopreservation. Blood and tissue samples were stored till further analysis. The lungs and spleen were fixed in 10% neutral buffered formalin for histological analysis. The test groups, the time points of necropsy and the number of mice used are shown in Table 1.

**Table 1:** The group number of mice used in the experiment and the time points of necropsy

Groups	Normal control	PCV2 infection control	PCV2 + combined high dose	PCV2 + combined medium dose	PCV2 + combined low dose	PCV2 + Matrine	PCV2 + Osthole	PCV2 + Ribavirin control
**Medication 0 d**	5	5	/	/	/	/	/	/
**Medication 3 d**	6	6	6	6	6	6	6	6
**Medication 6 d**	6	6	6	6	6	6	6	6
**Medication 9 d**	6	6	6	6	6	6	6	6
**Total mice**	23	23	18	18	18	18	18	18
	154							

### PCR and qPCR analysis

PCV2 DNA was respectively extracted according to the instructions of the blood/cell/tissue DNA extraction kit (TianGen, Beijing, China) from blood, liver and other tissues of PCV2 infected mice 5 d. PCV2 DNA concentration was determined by a nucleic acid concentration analyzer (NanoDrop Technologies, Wilmington, DE, USA).

PCR was performed for liver, thymus, spleen, lymph nodes, lung and blood samples collected from mice at 5 dpi. The expression of the *Cap* gene was detected by polymerase chain reaction (PCR) with primers 5’ GTC TAC ATT TCC AGC AGT TTG and 5’CTC CCG CCA TAC CAT AA. The 148 bp PCR product was analyzed by agarose gel electrophoresisand gel imaging were captured using gel imaging system (Azure Biosystems, Dublin, USA).

The PCV2 DNA of the mice liver was extracted after PCV2 infection at 5 d, 8 d, 11 d and 14 d, respectively and the DNA concentration was measured. The quantitative real-time PCR (qPCR) was applied to amplify the PCV2 *Cap* gene according to the 2 × SYBR Green qPCR Master Mix SYBR (Low ROX, Biotool, USA). Thermal cycling and fluorescence detection were conducted using Applied Biosystems®7500 real-time PCR system. Standard curve of generate by recombinant plasmid vectors containing PCV2 *Cap* gene fragments was used.

### Hematoxylin–eosin(HE)and Immunohistochemical(IHC)

The Bouin’s fixed and paraffin wax embedded lungs and spleens were cut into 4 µm sections. HE staining was performed according to standard protocols and the pathological changes of the lung and spleen tissues were examined.

We performed cleaved caspase-3 immunohistochemistry from paraffin sections to detect cell apoptosis using SP Rabbit&Mouse HRP Kit (DAB, CWBIO, Beijing, China). Cleaved caspase-3 monoclonal antibody was incubated for overnight at 4℃. Biotin-labeled secondary antibody working solution and HRP-labeled streptavidin were incubated at room temperature for 10 min, respectively. Visualization was with diaminobenzoate (DAB) according to standard protocol. The expression of cleaved caspase-3 protein in the spleen tissue was observed by microscope to assess the presence and distribution of the antigen. The equipment used for HE image and IHC image acquisition was(DM3000) (objective lenses: HC PL FLUOTAR 40X/0.8). These micrographs were photographed at a resolution of 10,667˟8000 with the cameras of DFC450C, and the image acquisition was obtained using LAS X software, which enhanced the images to 300 dpi. The lamp used for transmitted light BF- contrast imaging was 4W LED illumination.

### Measurement of body weight gain rate and viscera index

Body weight of mice in nomal control group and PCV2 infection group were recorded daily. The mice were dissected at 5 d after PCV2 infection and the lung and spleen were weighed to calculate the organ index. These visceras indices were calculated according to the following formula: Viscera index (%) = organ weight (g)/body weight (g) × 100; Body weight gain rate (%) = (body weight before necropsy-body weight before infection) (g)/body weight before infection (g) × 100.

### Western blot analysis

The total protein was extracted from the liver and spleen of the mice and the concentration was determined using Beyotime Biotechnology kit (Beyotime Biotechnology, Jiangsu, China). We performed western blotting using the following protocol: 40 μg proteins from each sample was separated on 15% SDS-PAGE, the SDS-PAGE gel was cut according to the KD of target proteins and comparison with the marker, the cropped gel was then transferred onto a polyvinylidene fluoride (PVDF) membrane (Millipore, USA) [13, 35]. The membrane was blocked for 2 h (hour) with 5% skim milk and then incubated with the primary antibody at 4 °C overnight. The membrane was incubated with the secondary antibody at room temperature for 1.5 h [13]. The manufacturer of primary and secondary antibodies and the dilution ratio used see antibody information in the methods section. The proteins levels of PCV2 Cap, GAPDH, GRP78, PERK, p-PERK, eIF2α, p-eIF2α, ATF4, CHOP, Bcl-2, Bax and cleaved caspase-3 were detected using an eECL Western Blot detection kit (Cwbio Inc, Beijing, China) and chemiluminescence imaging system (BIO-RAD, California, USA), quantified with Image J software (National Institutes of Health, Bethesda, MD, USA), respectively [13, 35].

### Statistical analysis

Data were expressed as the Mean ± Standard Errors Mean (SEM) of at least 3 repeated experiments. In this study, differences between the two groups were analyzed using t-test in GraphPad Prism ™ 5.0 Software (Inc. California, USA), *p˂0.05, ** *p*˂0.01, ***p˂0.001. “Bonferroni: Compare all pairs of columns” of One-way ANOVA was used for the analysis of difference between multiple groups. The gray intensity of protein bolts was analyzed by Image J software. Different letters (a, b, c, d, etc.) indicated significant difference between groups (*p*˂0.05).

## Supplementary Information

**Additional file 1.** 

**Additional file 2.** 

**Additional file 3.** 

**Additional file 4.** 

**Additional file 5.** 

**Additional file 6.** 

**Additional file 7.** 

**Additional file 8.**

## Data Availability

The datasets generated or analysed during the current study are available in this published article and its supplementary information files, and the sequencing data of PCV2-SH strain Cap gene have been deposited in the NCBI Sequence repository, and the gene number is GenBank: AY686763.1. The datasets used and analyzed during the current study are available from the corresponding author on reasonable request.

## Abbreviations

Dpi: Days post-infection
GRP78: Glucose-regulated protein 78
PCV2: Porcine circovirus type 2
PERK: Protein kinase RNA(PKP)-like extracellular regulated kinase
qPCR: Real time quantitative polymerase chain reaction
