# Supplementary material for: Matrine combined with Osthole inhibited the PERK apoptosis of splenic lymphocytes in PCV2-infected mice model
Source: BMC Vet Res. 2023 Jan 30;19:26. doi: 10.1186/s12917-023-03581-9 (PMC9885934; doi:10.1186/s12917-023-03581-9)
Supplement: Supplementary file 1 — Additional file 1. [file 12917_2023_3581_MOESM1_ESM.docx]

**Matrine combined with Osthole inhibited the PERK apoptosis of splenic lymphocytes in PCV2-infected mice model**

Yinlan Xu^1,2#^, Shuangxiu Wan^1,6#^, Panpan Sun^3^, Ajab Khan^1^, Jianhua Guo^4^, Xiaozhong Zheng^5^, Yaogui Sun^1^, Kuohai Fan^3^, Wei Yin^1^, Hongquan Li^1^ and Na Sun^1*^

**#These authors contributed equally to this work.**

***Corresponding author: Na Sun**：E-mail: [snzh060511@126.com](mailto:snzh060511@126.com)

College of Veterinary Medicine, Shanxi Agricultural University, Taigu, Shanxi 030801 China.

**The original PCR and HE staining images of Fig. 2**

**a** The original PCR images of Fig. 2a

**
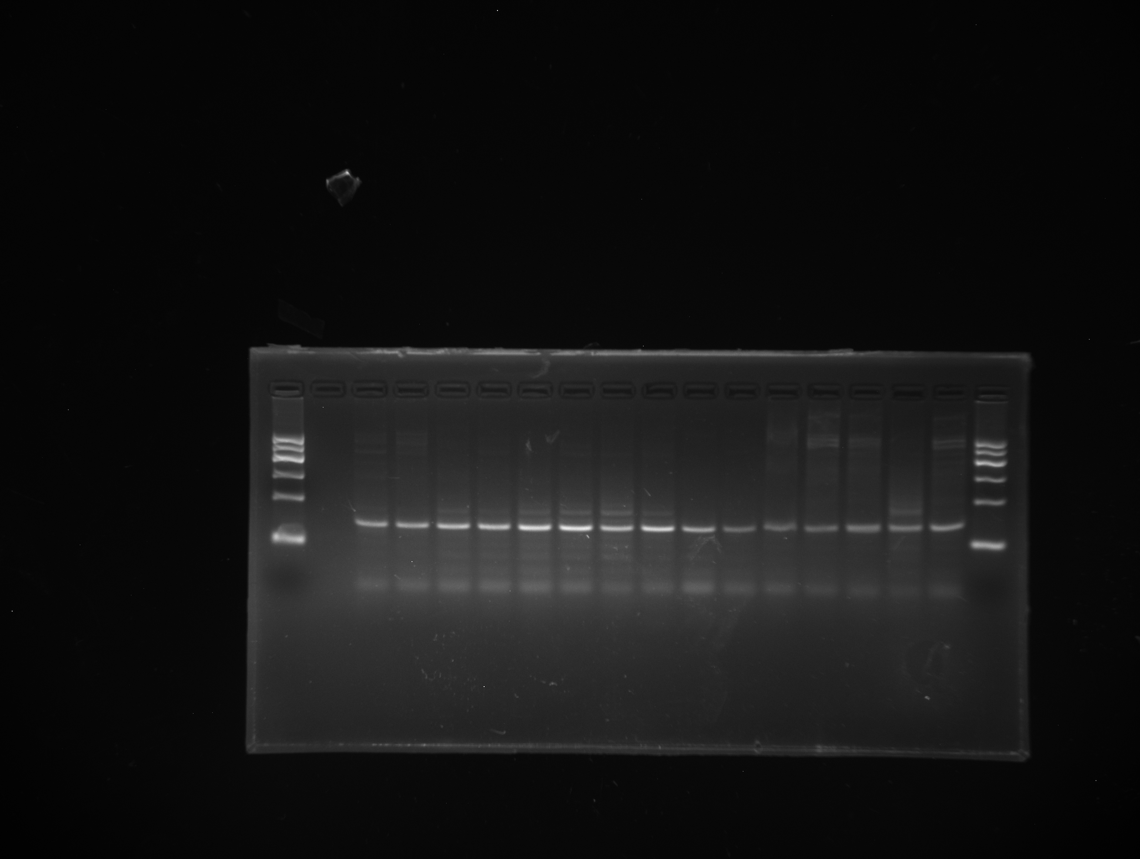
**

**200 bp**

**100 bp**

Spleen

Thymus

Liver

M K 1 2 3 4 5 1 2 3 4 5 1 2 3 4 5 M

**
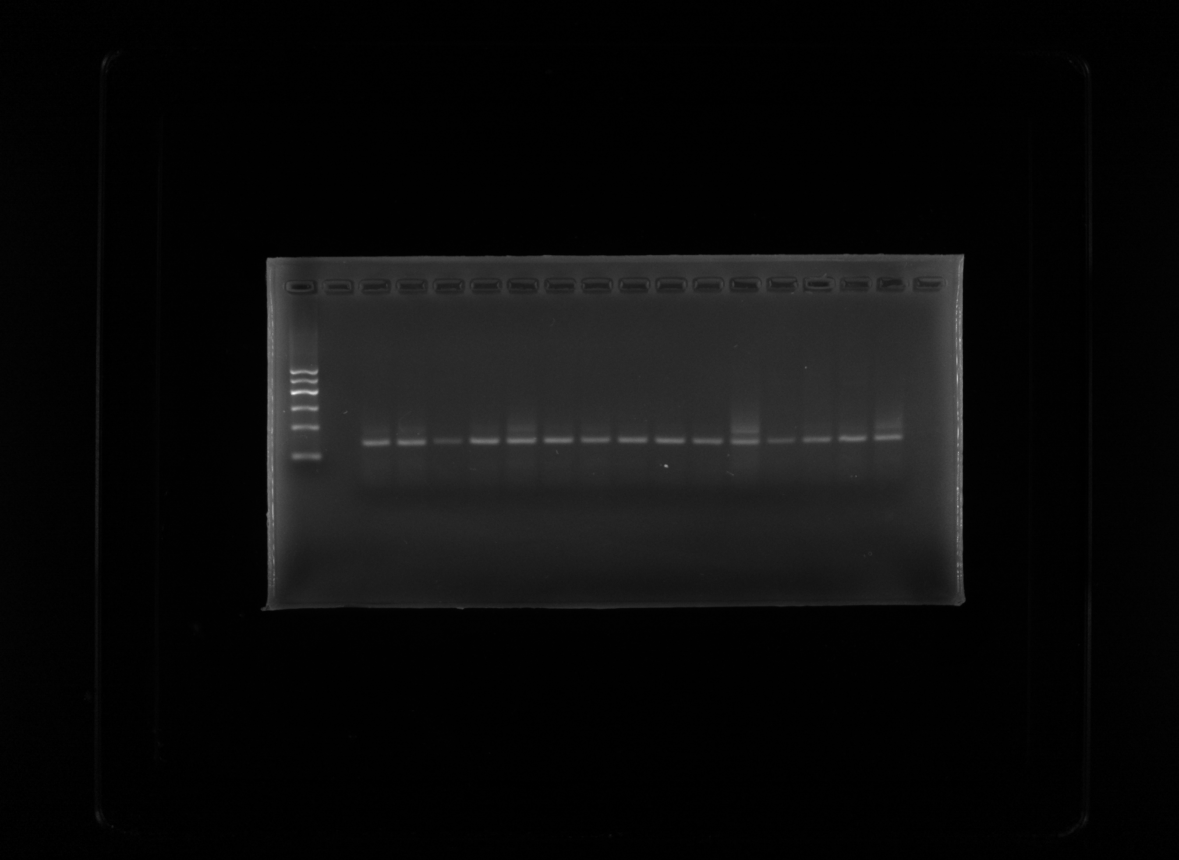
**

M K 1 2 3 4 5 1 2 3 4 5 1 2 3 4 5

Blood

Lung

Lymph nodes

**200 bp**

**100 bp**

**b** The original HE staining images of Fig. 2b

Normal group

**
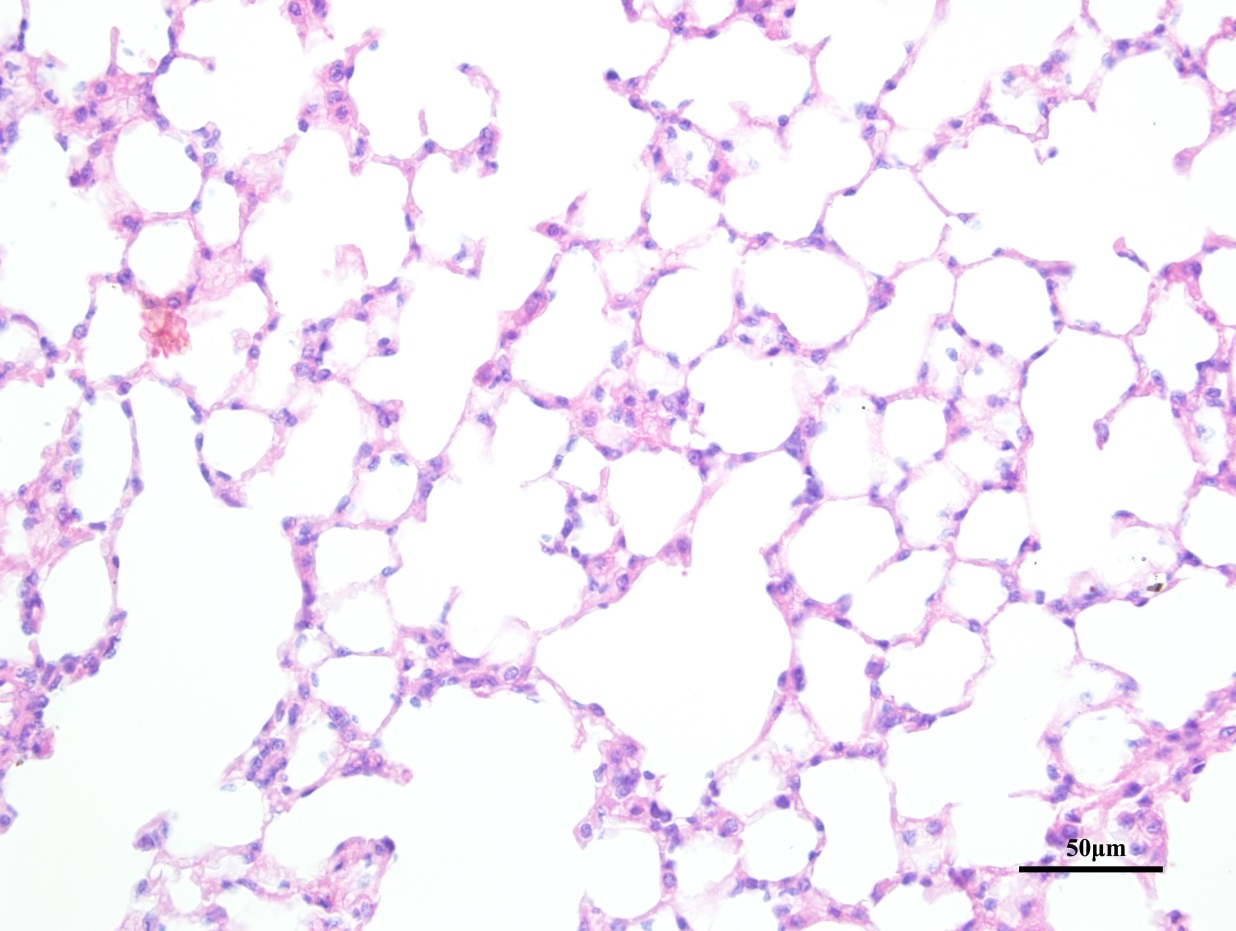
**

PCV2 group

**
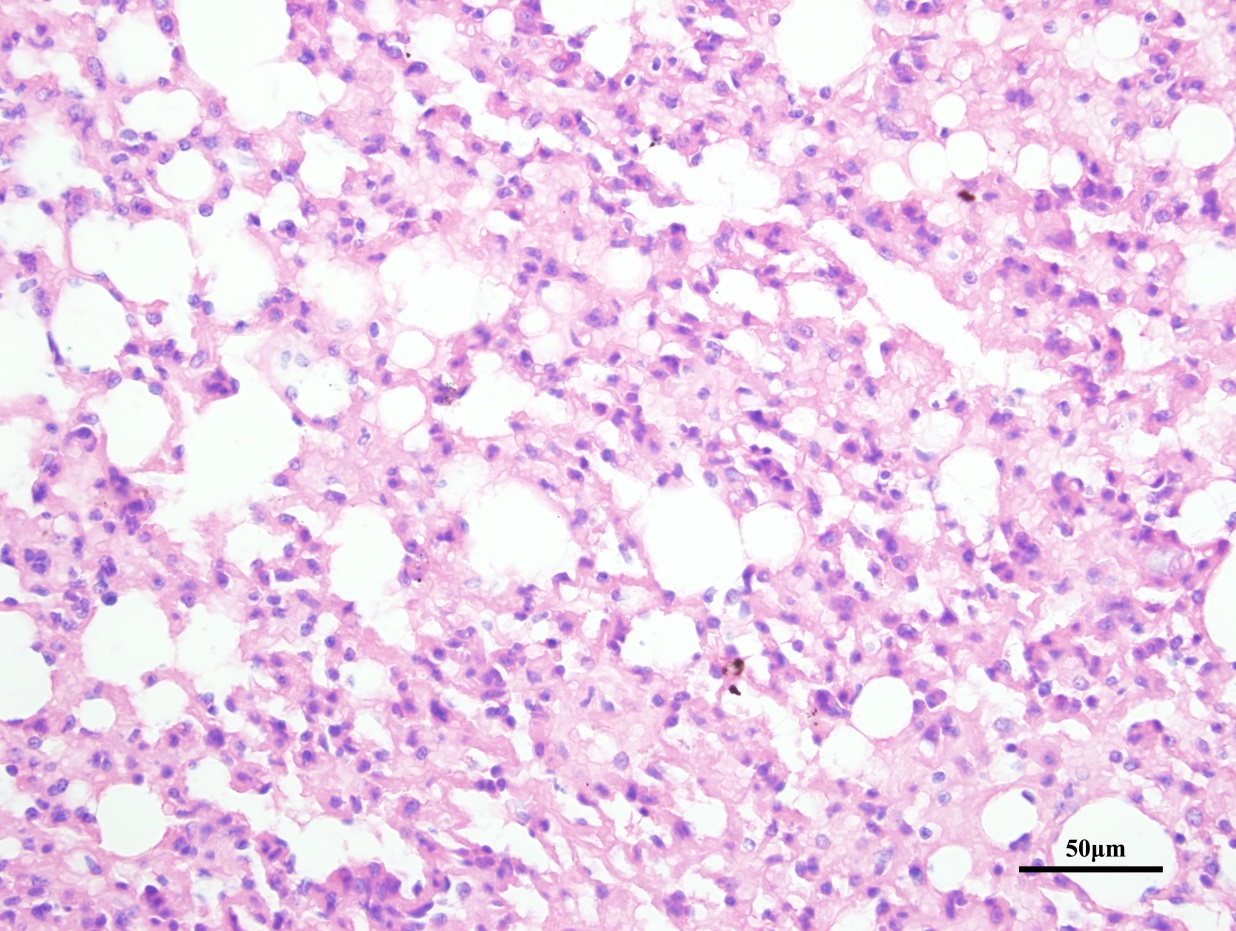
**
