# Supplementary material for: Matrine combined with Osthole inhibited the PERK apoptosis of splenic lymphocytes in PCV2-infected mice model
Source: BMC Vet Res. 2023 Jan 30;19:26. doi: 10.1186/s12917-023-03581-9 (PMC9885934; doi:10.1186/s12917-023-03581-9)

**Matrine combined with Osthole inhibited the PERK apoptosis of splenic lymphocytes in PCV2-infected mice model**

Yinlan Xu^1,2#^, Shuangxiu Wan^1,6#^, Panpan Sun^3^, Ajab Khan^1^, Jianhua Guo^4^, Xiaozhong Zheng^5^, Yaogui Sun^1^, Kuohai Fan^3^, Wei Yin^1^, Hongquan Li^1^ and Na Sun^1*^

**#These authors contributed equally to this work.**

***Corresponding author: Na Sun**：E-mail: [snzh060511@126.com](mailto:snzh060511@126.com)

College of Veterinary Medicine, Shanxi Agricultural University, Taigu, Shanxi 030801 China.

**Original IHC image of Fig. 6a**

**a** Normal group


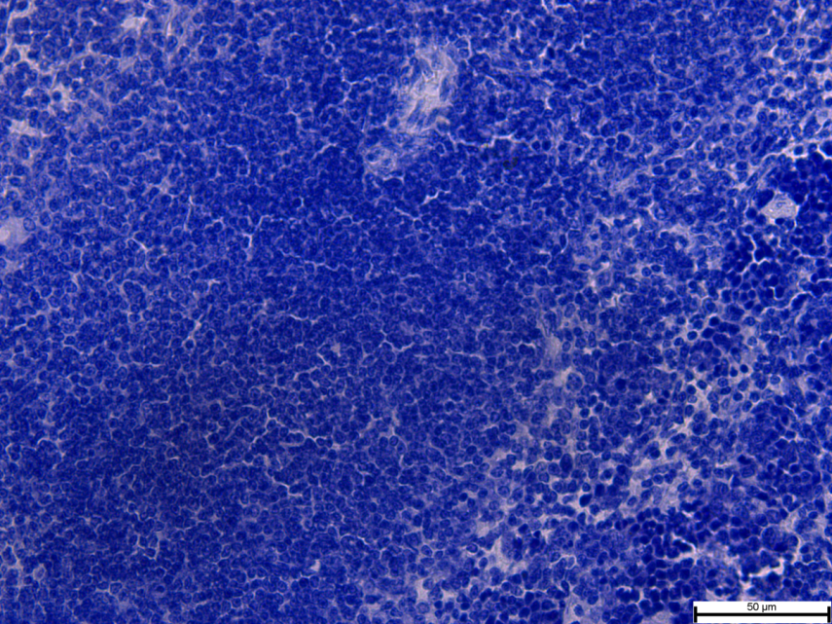


**b** PCV2 group


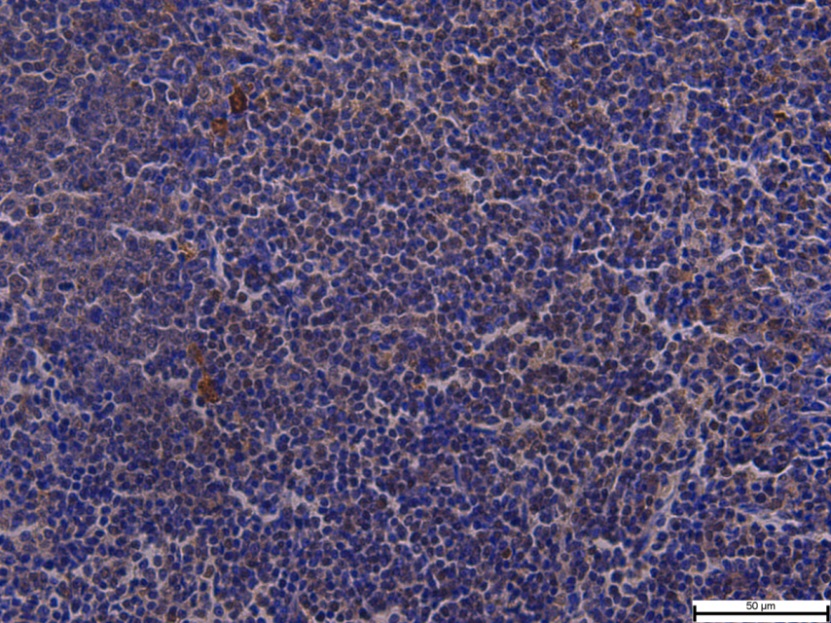


**c** High dose (40+12) mg/kg group


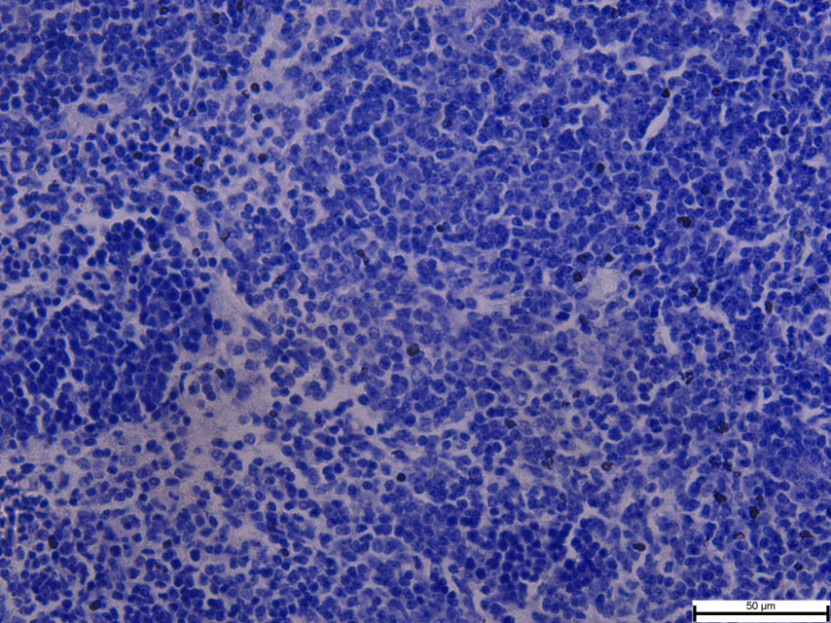


**d** Middle dose (20+6) mg/kg group


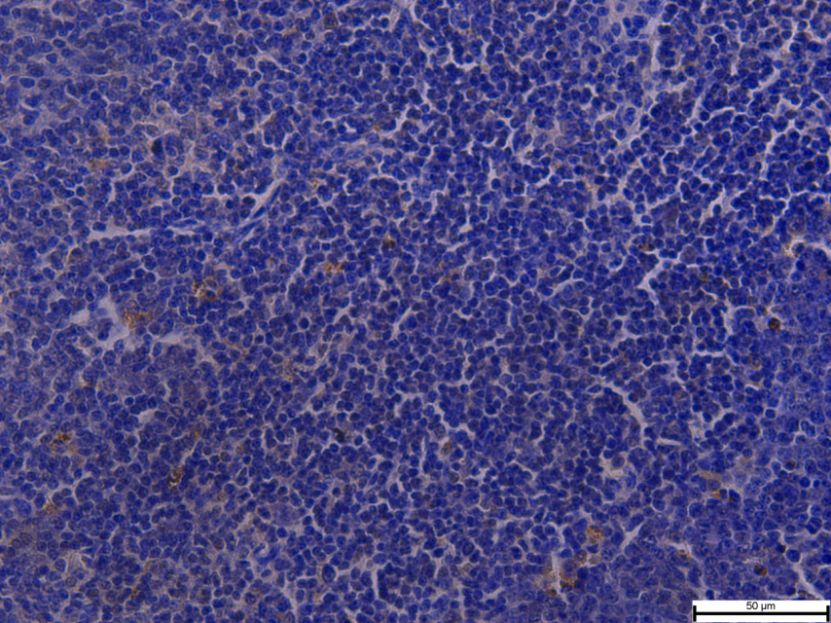


**e** Low dose (10+3) mg/kg group


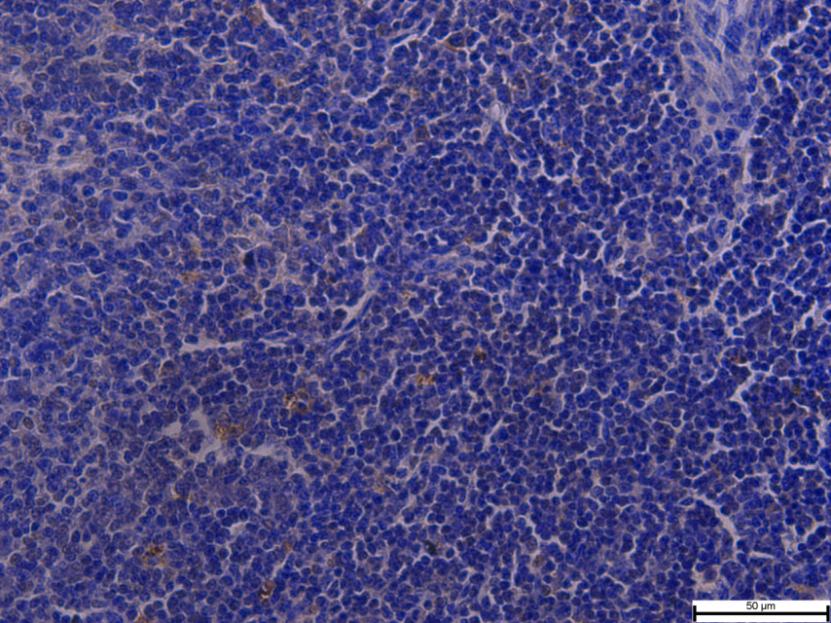


**f** Matrine 40 mg/kg group


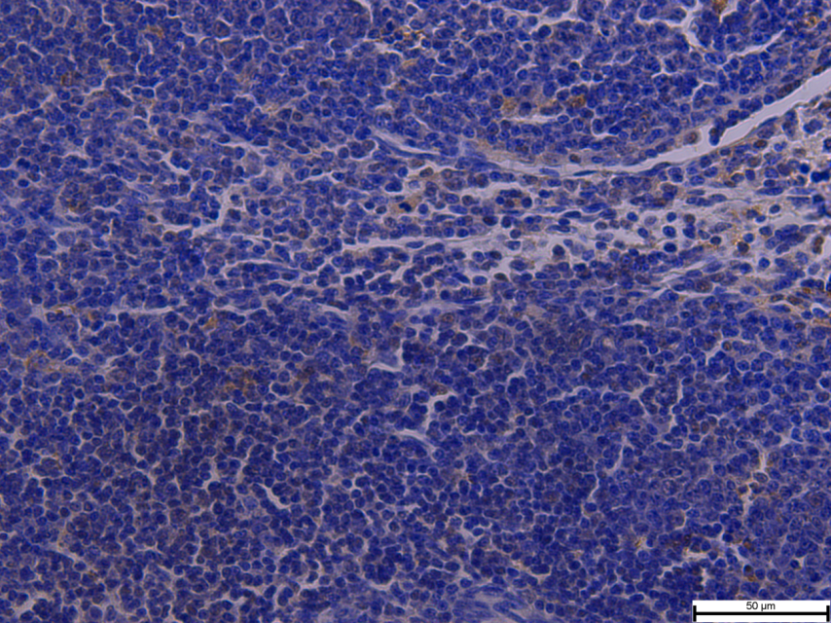


**g** Osthole 12 mg/kg group


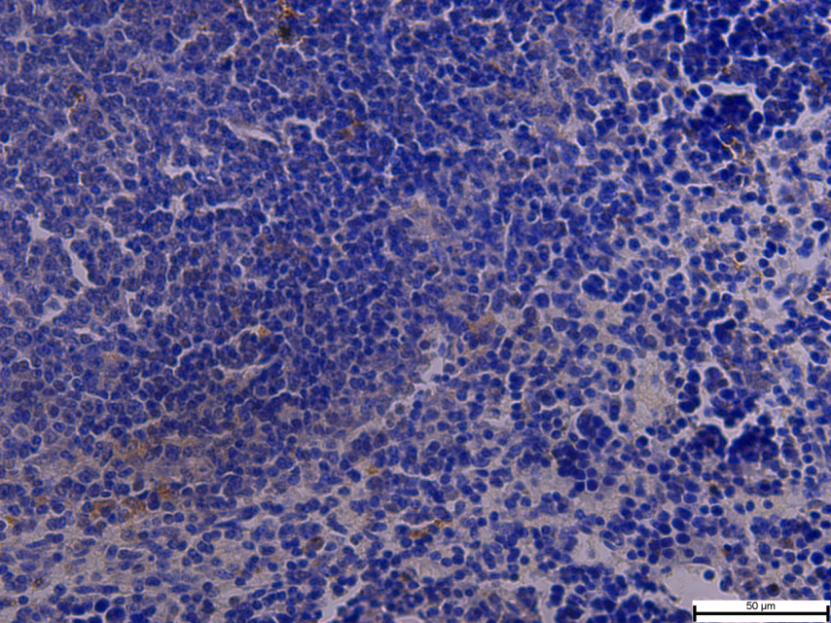


**h** Ribavirin 40 mg/kg group


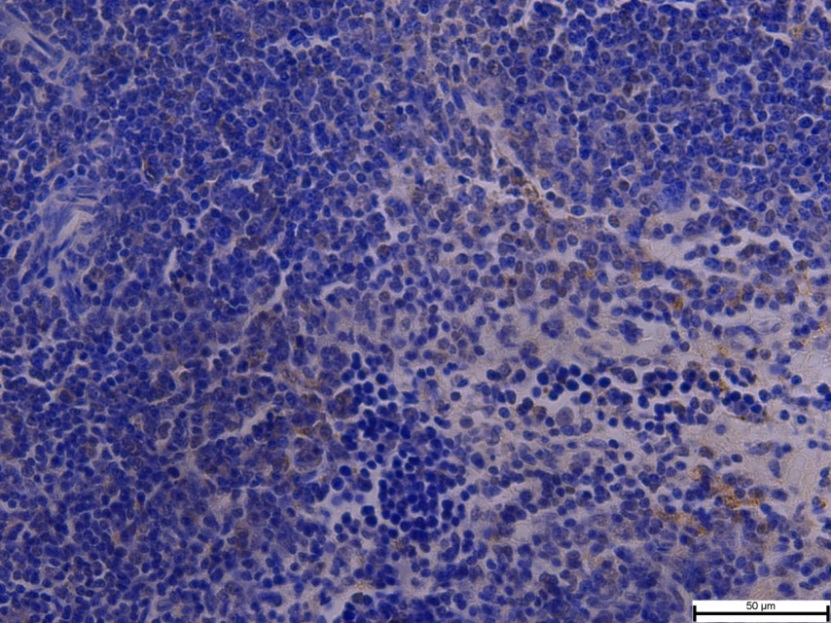

Supplement: Supplementary file 5 — Additional file 5. [file 12917_2023_3581_MOESM5_ESM.docx]
