# Supplementary material for: Matrine combined with Osthole inhibited the PERK apoptosis of splenic lymphocytes in PCV2-infected mice model
Source: BMC Vet Res. 2023 Jan 30;19:26. doi: 10.1186/s12917-023-03581-9 (PMC9885934; doi:10.1186/s12917-023-03581-9)

**Matrine combined with Osthole inhibited the PERK apoptosis of splenic lymphocytes in PCV2-infected mice model**

Yinlan Xu^1,2#^, Shuangxiu Wan^1,6#^, Panpan Sun^3^, Ajab Khan^1^, Jianhua Guo^4^, Xiaozhong Zheng^5^, Yaogui Sun^1^, Kuohai Fan^3^, Wei Yin^1^, Hongquan Li^1^ and Na Sun^1*^

**#These authors contributed equally to this work.**

***Corresponding author: Na Sun**：E-mail: [snzh060511@126.com](mailto:snzh060511@126.com)

College of Veterinary Medicine, Shanxi Agricultural University, Taigu, Shanxi 030801 China.

**Original blot image of 6b**

**a** Cleaved-caspase 3


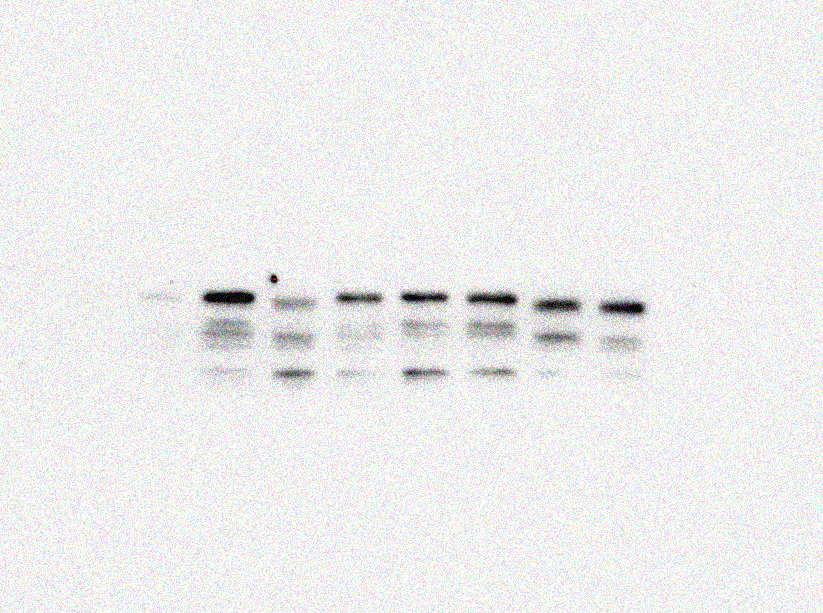


**Cleaved caspase-3**

**(32KDa)**

**PCV2 group**

**Normal control**

**High group (10+3)mg/kg**

**Matrine 40 mg/kg**

**Low group (40+12)mg/kg**

**Ribavirin 40 mg/kg**

**Middle group (20+6)mg/kg**

**Osthole 12 mg/kg**

**b** Bax


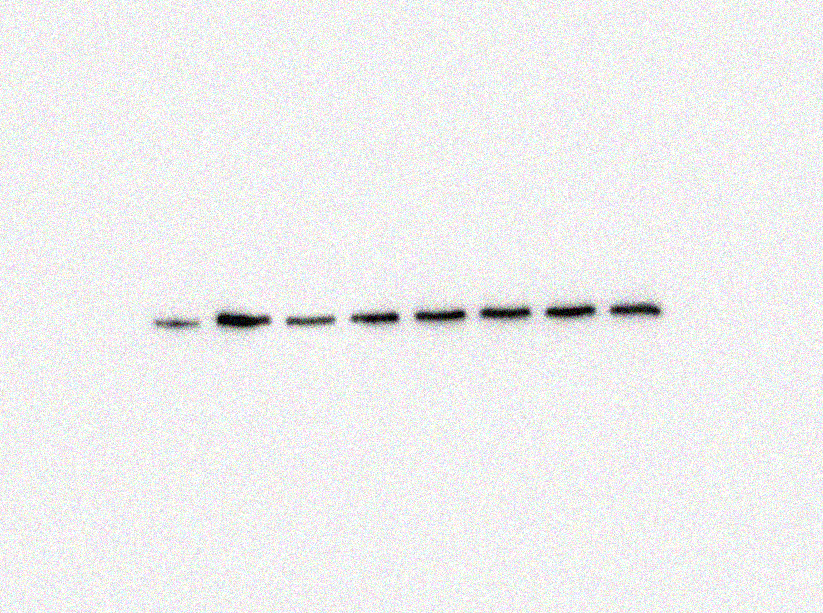


**Bax**

**(21KDa)**

**PCV2 group**

**Normal control**

**High group (10+3)mg/kg**

**Matrine 40 mg/kg**

**Low group (40+12)mg/kg**

**Ribavirin 40 mg/kg**

**Middle group (20+6)mg/kg**

**Osthole 12 mg/kg**

**c** Bcl-2


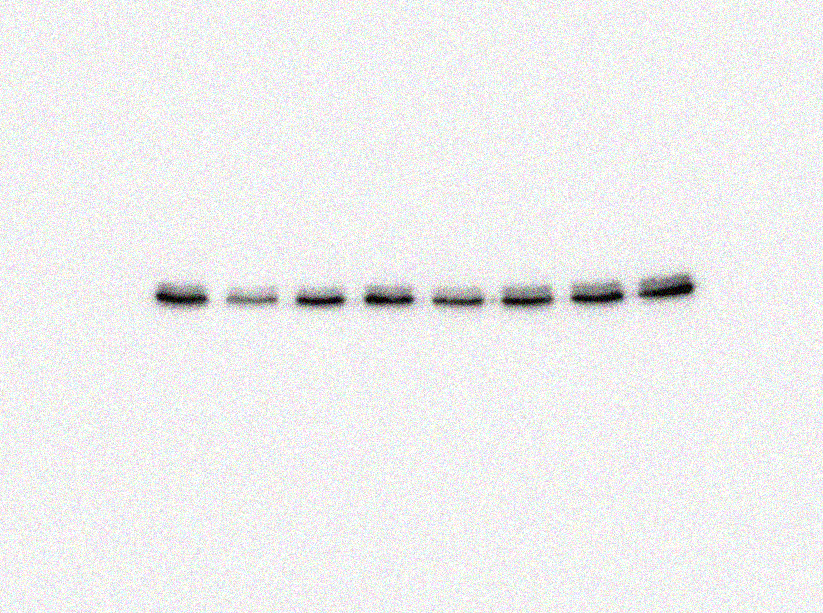


**Bcl-2**

**(26KDa)**

**PCV2 group**

**Normal control**

**High group (10+3)mg/kg**

**Matrine 40 mg/kg**

**Low group (40+12)mg/kg**

**Ribavirin 40 mg/kg**

**Middle group (20+6)mg/kg**

**Osthole 12 mg/kg**

**d** GAPDH

**GAPDH**

**(36KDa)**

**PCV2 group**

**Normal control**

**High group (10+3)mg/kg**

**Matrine 40 mg/kg**

**Low group (40+12)mg/kg**

**Ribavirin 40 mg/kg**

**Middle group (20+6)mg/kg**

**Osthole 12 mg/kg**


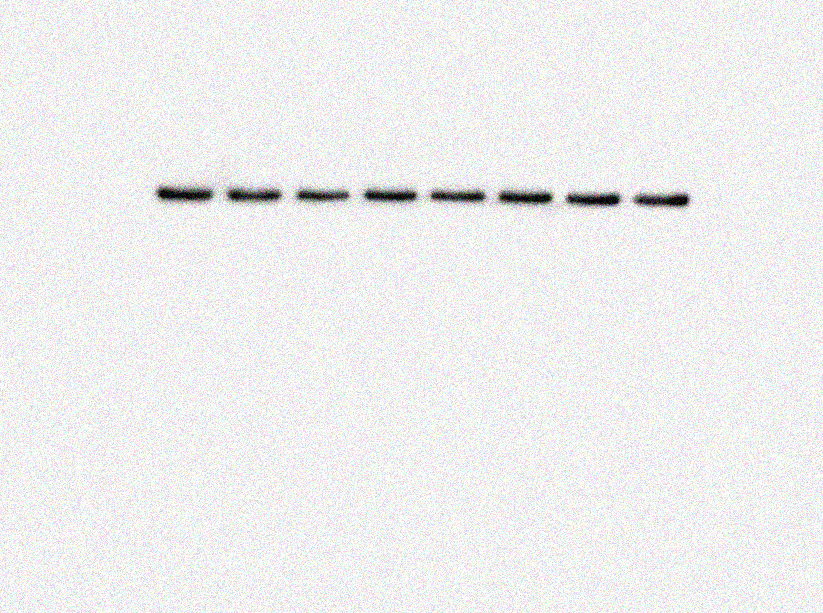

Supplement: Supplementary file 6 — Additional file 6. [file 12917_2023_3581_MOESM6_ESM.docx]
