# Supplementary material for: Matrine combined with Osthole inhibited the PERK apoptosis of splenic lymphocytes in PCV2-infected mice model
Source: BMC Vet Res. 2023 Jan 30;19:26. doi: 10.1186/s12917-023-03581-9 (PMC9885934; doi:10.1186/s12917-023-03581-9)
Supplement: Supplementary file 7 — Additional file 7. [file 12917_2023_3581_MOESM7_ESM.docx]

**Matrine combined with Osthole inhibited the PERK apoptosis of splenic lymphocytes in PCV2-infected mice model**

Yinlan Xu^1,2#^, Shuangxiu Wan^1,6#^, Panpan Sun^3^, Ajab Khan^1^, Jianhua Guo^4^, Xiaozhong Zheng^5^, Yaogui Sun^1^, Kuohai Fan^3^, Wei Yin^1^, Hongquan Li^1^ and Na Sun^1*^

**#These authors contributed equally to this work.**

***Corresponding author: Na Sun**：E-mail: [snzh060511@126.com](mailto:snzh060511@126.com)

College of Veterinary Medicine, Shanxi Agricultural University, Taigu, Shanxi 030801 China.

**Original blot image of 7A and 7b**

**a** GRP78 of Fig. 7a


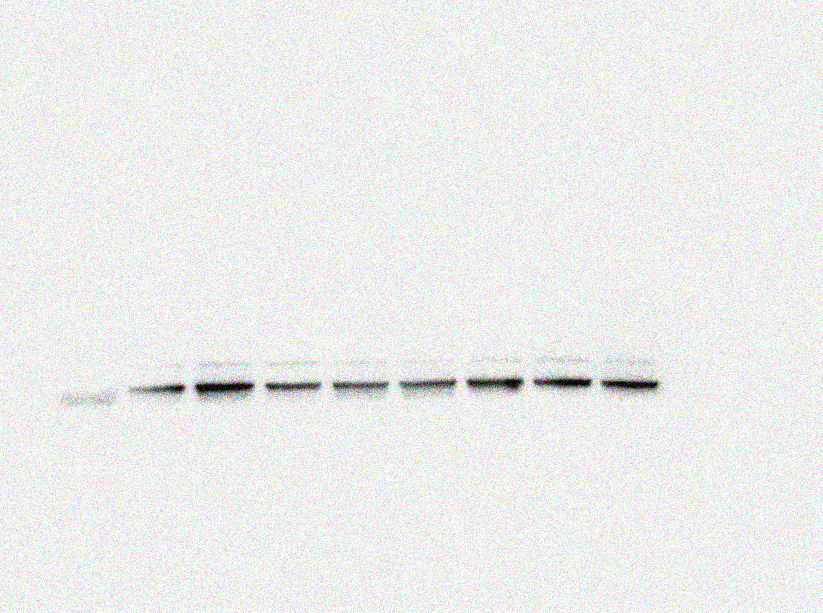


**GRP78**

**(78KDa)**

**PCV2 group**

**Normal control**

**High group (10+3)mg/kg**

**Matrine 40 mg/kg**

**Low group (40+12)mg/kg**

**Ribavirin 40 mg/kg**

**Middle group (20+6)mg/kg**

**Osthole 12 mg/kg**

**b** p^-PERK^ of Fig. 7a


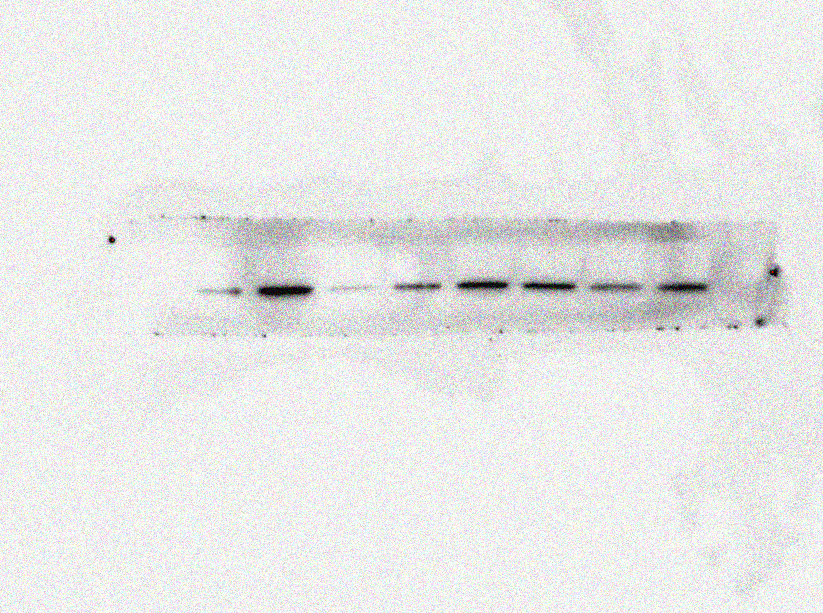


**p^-PERK^ (150KDa)**

**PCV2 group**

**Normal control**

**High group (10+3)mg/kg**

**Matrine 40 mg/kg**

**Low group (40+12)mg/kg**

**Ribavirin 40 mg/kg**

**Middle group (20+6)mg/kg**

**Osthole 12 mg/kg**

**c** t^-PERK^ of Fig. 7a


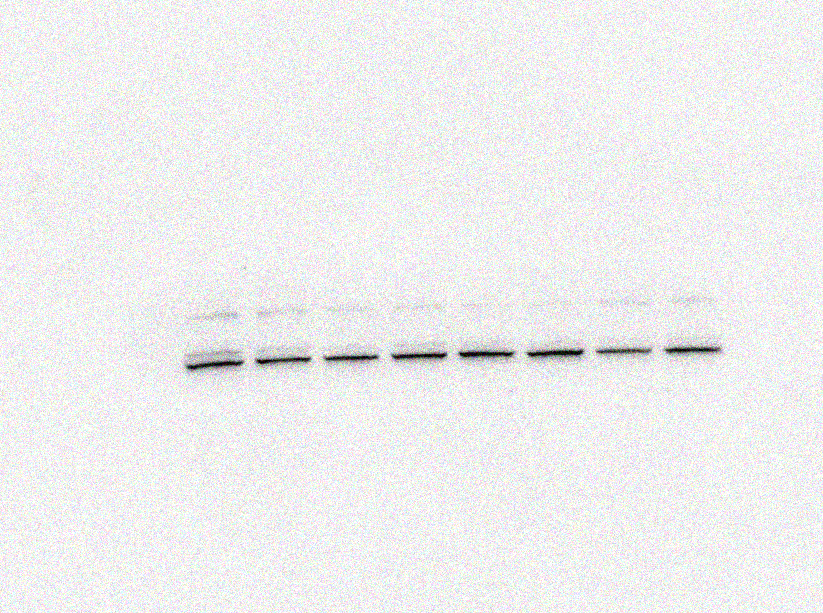


**t^-PERK^ (150KDa)**

**PCV2 group**

**Normal control**

**High group (10+3)mg/kg**

**Matrine 40 mg/kg**

**Low group (40+12)mg/kg**

**Ribavirin 40 mg/kg**

**Middle group (20+6)mg/kg**

**Osthole 12 mg/kg**

**d** GAPDH of Fig. 7a


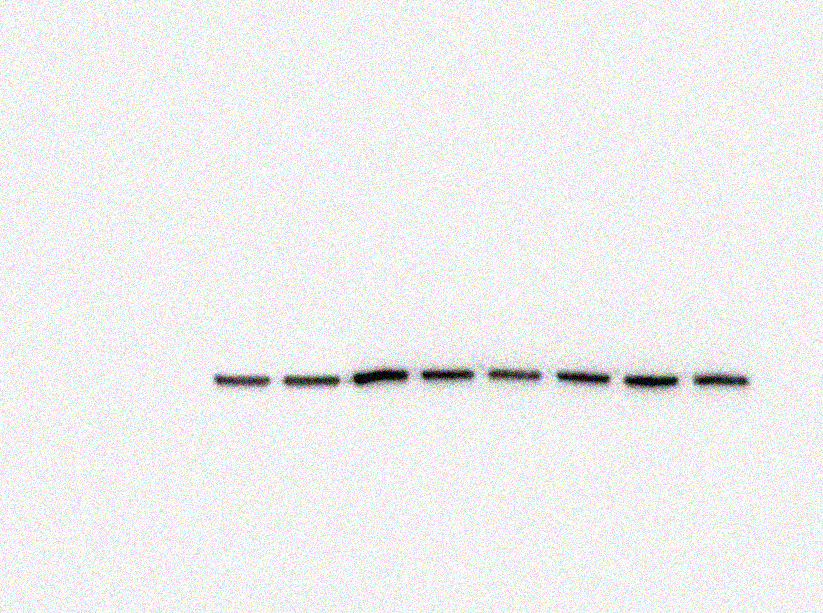


**GAPDH**

**(36KDa)**

**PCV2 group**

**Normal control**

**High group (10+3)mg/kg**

**Matrine 40 mg/kg**

**Low group (40+12)mg/kg**

**Ribavirin 40 mg/kg**

**Middle group (20+6)mg/kg**

**Osthole 12 mg/kg**

**a** p^-eIF2α^ of Fig. 7b


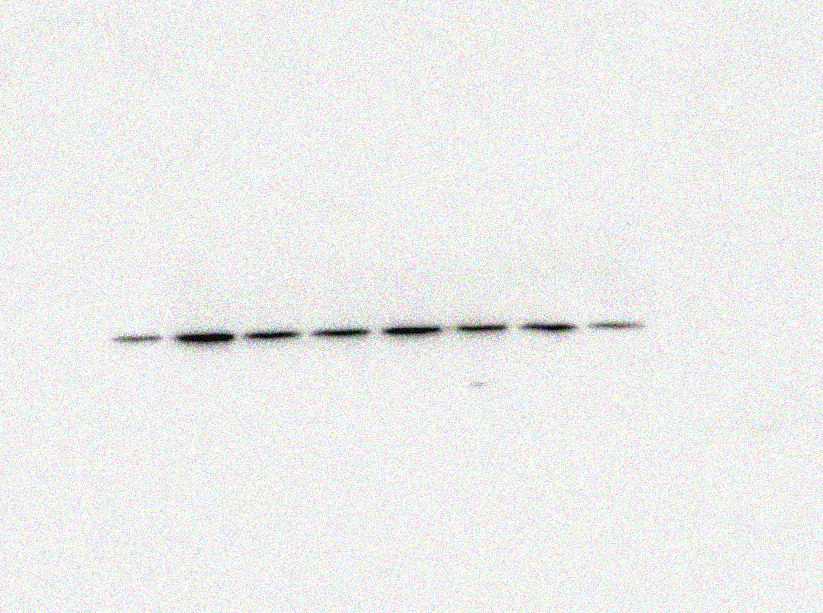


**P^-eIF2α^**

**(36KDa)**

**PCV2 group**

**Normal control**

**High group (10+3)mg/kg**

**Matrine 40 mg/kg**

**Low group (40+12)mg/kg**

**Ribavirin 40 mg/kg**

**Middle group (20+6)mg/kg**

**Osthole 12 mg/kg**

**b** t^-eIF2α^ of Fig. 7b

**t^-eIF2α^**

**(65KDa)**

**PCV2 group**

**Normal control**

**High group (10+3)mg/kg**

**Matrine 40 mg/kg**

**Low group (40+12)mg/kg**

**Ribavirin 40 mg/kg**

**Middle group (20+6)mg/kg**

**Osthole 12 mg/kg**


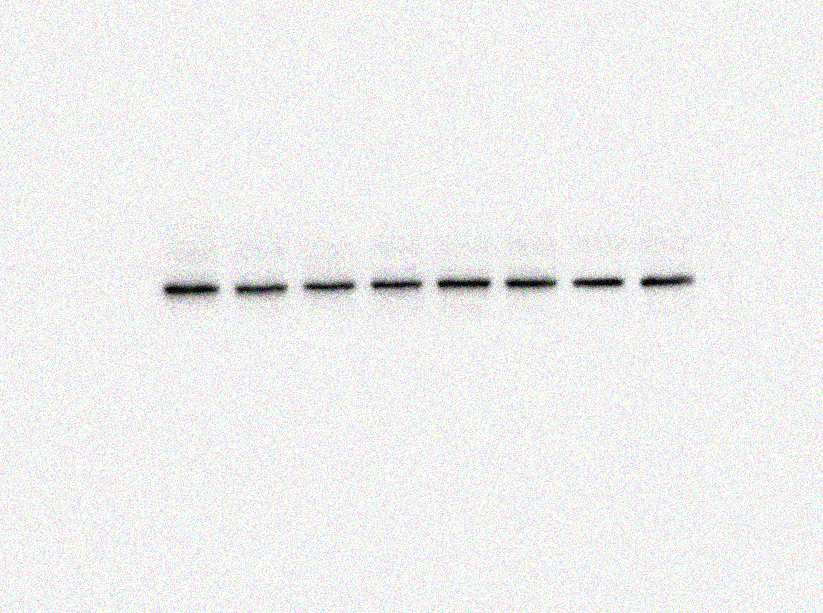


**c** ATF4 of Fig. 7b


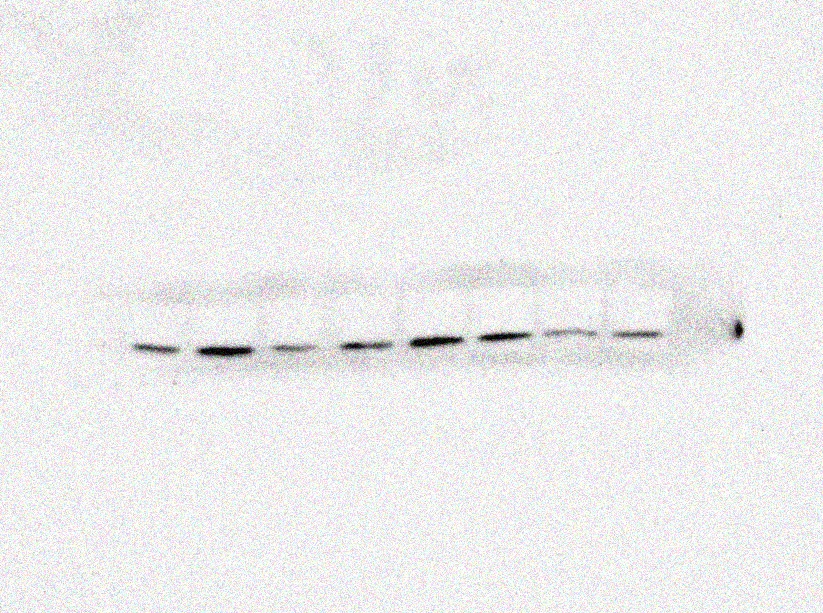


**ATF4**

**(76KDa)**

**PCV2 group**

**Normal control**

**High group (10+3)mg/kg**

**Matrine 40 mg/kg**

**Low group (40+12)mg/kg**

**Ribavirin 40 mg/kg**

**Middle group (20+6)mg/kg**

**Osthole 12 mg/kg**

**d** CHOP of Fig. 7b

**CHOP**

**(27KDa)**

**PCV2 group**

**Normal control**

**High group (10+3)mg/kg**

**Matrine 40 mg/kg**

**Low group (40+12)mg/kg**

**Ribavirin 40 mg/kg**

**Middle group (20+6)mg/kg**

**Osthole 12 mg/kg**


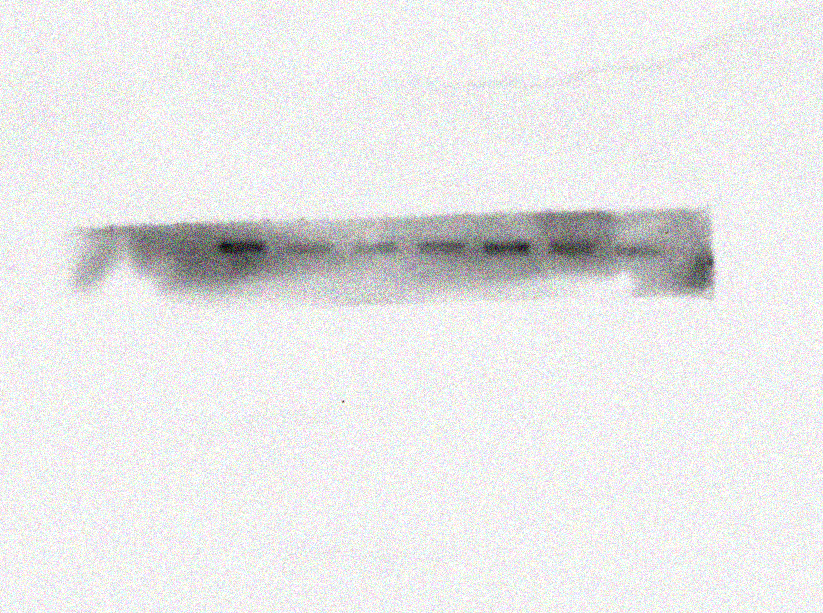


**e** GAPDH of Fig. 7b


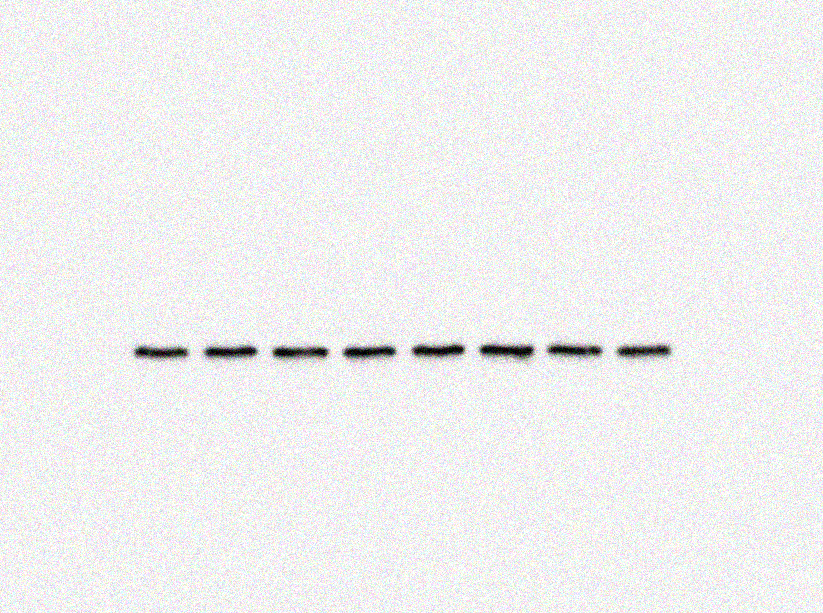


**GAPDH**

**(36KDa)**

**PCV2 group**

**Normal control**

**High group (10+3)mg/kg**

**Matrine 40 mg/kg**

**Low group (40+12)mg/kg**

**Ribavirin 40 mg/kg**

**Middle group (20+6)mg/kg**

**Osthole 12 mg/kg**
